# Low CT-Derived Thoracic Muscle Area and Composite Weaning Failure in Mechanically Ventilated ICU Patients with Pneumonia: An Exploratory Analysis by Age Group

**DOI:** 10.3390/jcm15156105

**Published:** 2026-08-05

**Authors:** Seonghye Jung, Kwonhyung Hyung, Heemoon Park, Hyun Woo Lee, Jung-Kyu Lee, Tae Yun Park, Eun Young Heo, Deog Kyeom Kim, Hyo Jin Lee

**Affiliations:** 1 Division of Pulmonary and Critical Care Medicine, Department of Internal Medicine, College of Medicine, Seoul National University, Seoul 03080, Republic of Korea; 2 Division of Respiratory and Critical Care, Department of Internal Medicine, Seoul Metropolitan Government—Seoul National University Boramae Medical Center, Seoul 07061, Republic of Korea; 3 Department of Internal Medicine, College of Medicine, Seoul National University, Seoul 03080, Republic of Korea

**Keywords:** low thoracic muscle area, ventilator weaning, pneumonia, CT scan, age factors

## Abstract

**Background/Objectives:** Whether the prognostic contribution of low skeletal muscle mass varies with age in ventilated patients is untested. We assessed whether the association between low computed tomography (CT)-derived thoracic muscle area and ventilator-liberation outcomes differs by age. **Methods:** This single-center retrospective cohort included 607 adults with pneumonia requiring invasive ventilation in the intensive care unit (ICU) between 2016 and 2021. Thoracic muscle area was quantified by deep-learning segmentation of the whole thoracic musculature (first to twelfth thoracic vertebra) on the chest CT obtained closest to ICU admission and normalized to the length of the thoracic spine (median offset 0 days; 86% within ±7 days, before or after); low thoracic muscle area was the sex-specific lowest quartile. The primary outcome was composite weaning failure (extubation failure, tracheostomy, or ventilation >21 days). Multivariable logistic regression stratified at 75 years tested a muscle-area-by-age interaction. **Results:** Composite weaning failure occurred in 290 patients (47.8%). In fully adjusted complete-case models (*n* = 539), low thoracic muscle area was associated with composite weaning failure in younger patients (adjusted odds ratio 3.63; 95% CI 1.67–7.87) but not significantly in older patients (1.68; 0.91–3.13); the age interaction was not significant (*p* = 0.061) and was removed by height-normalization and by correction for multiple comparisons. A graded association across muscle-area quartiles was present in both strata (lowest vs. highest quartile: younger 4.79, older 2.53). Low thoracic muscle area was not associated with ICU mortality in either stratum, although an exploratory landmark analysis showed higher 7-day mortality in younger patients with low muscle area (20.6% vs. 10.8%). **Conclusions:** Low CT-derived thoracic muscle area was associated with composite weaning failure in both age strata, with a larger point estimate in patients younger than 75 years, although the age interaction was not significant in the fully adjusted model and was removed by height-normalization and by correction for multiple comparisons. The graded association in both age strata is hypothesis-generating and requires prospective validation.

## 1. Introduction

Pneumonia is the leading infectious cause of mortality worldwide, responsible for approximately 2.2 million deaths annually, with one in nine hospitalized patients requiring intensive care unit (ICU) admission [1,2]. A substantial proportion of these patients require invasive mechanical ventilation (MV); in the recent WEAN SAFE prospective study of 5869 mechanically ventilated patients across 50 countries, only 65% were successfully weaned at day 90 and ICU mortality reached 31.8%, underscoring that failure to achieve successful ventilator liberation, encompassing extubation failure, tracheostomy, and prolonged MV, is a major determinant of ICU length of stay, complications, and long-term functional outcomes [3,4]. Despite advances in lung-protective ventilation and weaning protocols, predicting which patients will fail ventilator liberation at ICU admission remains an unresolved clinical challenge.

Sarcopenia, defined as pathological loss of skeletal muscle mass, strength, and function, has been reported in approximately 43% of mechanically ventilated patients (95% CI 34–51%) [5] and is associated with adverse outcomes including prolonged MV, difficult weaning, and increased mortality [5,6,7]. Computed tomography (CT)-derived thoracic muscle quantification offers an objective, clinically pragmatic approach that requires no additional imaging beyond the diagnostic workup routinely performed for pneumonia [8,9,10]. Prior studies in surgical and mixed ICU populations have confirmed that CT-measured muscle depletion independently predicts difficult weaning and ICU mortality [6,8,11,12].

Precision of nomenclature matters here. The European Working Group on Sarcopenia in Older People (EWGSOP2) defines sarcopenia by low muscle mass together with low muscle strength and, for severe disease, low physical performance, and distinguishes primary sarcopenia, in which no cause other than aging is apparent, from secondary sarcopenia arising from disease, inactivity or inadequate nutrition [13]. In critically ill patients these categories overlap extensively with frailty, cachexia, protein–energy malnutrition and low physiological reserve, and the same CT appearance may be produced by any of them. CT-derived thoracic muscle area quantifies only the mass component of this construct; it cannot establish low strength or impaired performance, and it does not by itself separate primary from secondary sarcopenia or from concurrent cachexia or malnutrition. For this reason, we refer throughout to low CT-derived thoracic muscle area rather than to sarcopenia.

Previous studies, however, enrolled heterogeneous populations spanning a wide age range and did not formally examine whether the prognostic contribution of muscle depletion varies across the lifespan [5,6,7]. This is a clinically consequential gap, because the biological meaning of low thoracic muscle area differs fundamentally between younger and older patients. In younger patients, where cardiac, renal, and cognitive reserves are relatively preserved, low thoracic muscle area may represent the primary structural barrier to ventilator liberation, producing a clear prognostic signal against a background of otherwise intact physiology. In older patients, multimorbidity, immunosenescence, cognitive decline, and frailty create a constellation of competing adverse determinants that collectively dilute the independent contribution of any single factor, a ceiling effect that predicts attenuation of the low thoracic muscle area signal with advancing age [4,14,15,16]. Although this is an a priori hypothesis rather than a demonstrated mechanism, to our knowledge, no study has examined whether the association between CT-derived thoracic muscle area and weaning outcomes in ICU patients with pneumonia is modified by age.

We therefore aimed to determine whether CT-derived thoracic muscle area at ICU admission is independently associated with clinical outcomes in mechanically ventilated ICU patients with pneumonia, and whether its prognostic impact differs between younger and older patients.

## 2. Materials and Methods

### 2.1. Study Design and Participants

This was a single-center, retrospective observational cohort study conducted at a tertiary-care academic medical center. All adult patients (age ≥ 18 years) admitted to the medical and surgical ICUs with a primary diagnosis of severe pneumonia, irrespective of whether they subsequently required invasive MV, between January 2016 and December 2021 were screened for eligibility (*n* = 816).

Patients were eligible if they met all of the following inclusion criteria: (1) age ≥ 18 years; and (2) availability of a chest or thoracoabdominal CT scan suitable for thoracic muscle measurement. No minimum ICU length of stay was required, so that patients who died or were transferred early were retained. Patients were excluded if they had (1) a terminal illness with a do-not-resuscitate order documented at ICU admission, or (2) were admitted to the ICU primarily for a critical condition other than pneumonia.

### 2.2. CT Measurement of Thoracic Muscle Mass

Thoracic skeletal muscle was quantified on the chest CT scan obtained closest to the time of ICU admission. Eligibility required a measurable scan rather than a fixed time window: the median offset between CT and ICU admission was 0 days (IQR –1 to 0; full range −33 to +363), 86% of scans (521/607) were obtained within ±7 days, and timing was similar in patients with and without low thoracic muscle area (median 0 days in both; Mann–Whitney *p* = 0.078). Measurements used the automated segmentation pipeline previously developed and reported in this institutional cohort [17]. Initial three-dimensional segmentation of the thoracic musculature was performed on an analysis workstation (MEDIP, MEDICAL IP, Seoul, South Korea). Segmentation used a custom deep-learning model (U-Net and nnU-Net) trained to delineate the targeted muscle groups, and segmental muscle mass along the thoracic spine was quantified with dedicated artificial-intelligence software (DeepCatch, version 1.x.x.x; MEDICALIP, Seoul, South Korea) [18]. The segmentation network was trained on the chest CT scans of this institutional pneumonia cohort and tested externally on 343 chest CT scans from a second hospital, where it achieved mean Dice similarity coefficients of 0.92 (U-Net) and 0.94 (nnU-Net) against the reference segmentation [17]. The exposure variable was the mean thoracic muscle area (mm^2^), defined as the total segmented thoracic muscle volume divided by the craniocaudal length of the region of interest along the thoracic spine (first to twelfth thoracic vertebra; median 320 mm, interquartile range 293 to 347 mm) [17]. The segmented compartment comprised the bilateral pectoralis major, pectoralis minor, intercostal, subscapularis, infraspinatus, trapezius, and paraspinal muscle areas, identified by Hounsfield unit thresholds of −29 to +150 HU to isolate skeletal muscle tissue [19]. This normalization makes the measurement independent of scan coverage and of thoracic height; single-level cross-sectional areas at the fourth and twelfth thoracic vertebrae were also generated but were not used. The thoracic compartment was used because it captures the accessory respiratory musculature relevant to ventilator liberation, correlates with whole-body and lumbar (L3) skeletal muscle mass, [10], and is reliably visible on every chest CT obtained for pneumonia, whereas the L3 level is frequently outside the scanned field. The measurement is therefore a global thoracic muscle-area surrogate and not a measure of diaphragm mass or respiratory pump capacity, neither of which was assessed. Initial delineation on the workstation is semi-automatic and was performed under the guidance of an experienced radiologist, and the final segmentation was produced by the deep-learning model; the investigators who extracted the values were blinded to clinical outcomes. Because segmentation was standardized and largely automated, and the deep-learning component is deterministic, measurement variability is expected to be small, although a small degree of observer dependence at the workstation step cannot be entirely excluded. Formal intra- and inter-observer agreement was therefore not assessed in this cohort; segmentation accuracy is instead characterized by the external-test Dice similarity coefficients reported above, and is addressed further in Section 4.

### 2.3. Definition of Exposure

Low thoracic muscle area was used as a CT-derived surrogate for low muscle mass rather than as a clinical diagnosis of sarcopenia, and was defined as a total thoracic muscle cross-sectional area below the sex-specific lowest quartile, so that approximately one quarter of the cohort is exposed by construction. No validated normative CT thresholds exist for this population and lumbar indices are inapplicable without abdominal CT, so cut-offs were pre-specified from the full 778-patient CT cohort rather than from the 607 ventilated patients, in whom MV is outcome-adjacent and could induce selection bias. The cut-offs were 8729 mm^2^ for men and 6715 mm^2^ for women; the ventilated-cohort derivation was nearly identical (8844 and 6721 mm^2^; 4 of 607 patients reclassified, 0.7%). The exposure was defined on absolute area without normalization; height-indexed and alternative-threshold analyses are reported in Supplementary Information S1 and S2, Panel B.

### 2.4. Clinical Data Collection

Clinical and demographic data were extracted from the institutional electronic medical records system. Collected variables included age, sex, body mass index (BMI), admission diagnosis, clinical severity scores at ICU admission (APACHE II, SOFA, and SAPS-II), vital signs at admission (blood pressure, respiratory rate, body temperature), laboratory parameters (arterial blood gas analysis, complete blood count, C-reactive protein, creatinine, and lactate), pre-existing comorbidities (hypertension, diabetes mellitus, chronic obstructive pulmonary disease, interstitial lung disease, chronic kidney disease, chronic liver disease, congestive heart failure, cerebrovascular disease and/or dementia), ICU interventions (MV, high-flow nasal cannula, vasopressor use, hemodialysis, extracorporeal membrane oxygenation), and outcome data including MV duration, extubation success, reintubation, tracheostomy, and survival status with time to death. APACHE II scores were calculated from the worst recorded values within the first 24 h of ICU admission.

### 2.5. Outcomes

The primary outcome was composite weaning failure, defined as the occurrence of any of the following among mechanically ventilated patients: (1) extubation failure, defined as the need for reintubation within 48 h of planned extubation or failure to achieve planned extubation following a spontaneous breathing trial; (2) tracheostomy during the ICU stay; or (3) prolonged MV, defined as MV duration exceeding 21 days. The composite was evaluated at the patient level as the union of its three components: a patient meeting more than one component was counted once and no hierarchy was applied, so the 290 events comprised 390 component occurrences (extubation failure 211, tracheostomy 134, prolonged MV 45; full cross-classification in Supplementary Information S2, Panel C). Death was not a separate component: a patient who died while still receiving invasive MV had by definition not achieved planned extubation and therefore already met the extubation-failure component. The 132 patients (21.7%) who died without successful extubation were accordingly counted as events rather than excluded or censored, no minimum ICU length of stay was required, and no patient entered the composite by virtue of death alone. Because death is also a competing risk for liberation, liberation from MV was additionally analyzed in a competing-risk framework (Supplementary Information S6); because 36 patients died within 48 h of an extubation not followed by reintubation and were therefore classified as having achieved extubation, a sensitivity analysis counting these deaths as weaning failure is also reported (Supplementary Information S2, Panel C).

The composite was pre-specified as a pragmatic operational endpoint rather than a unified biological construct: its components share the consequence of failure to achieve timely ventilator liberation but differ in clinical severity and are not assumed to share a mechanism. A composite was required for adequate power in the pre-specified age-interaction test, because the least frequent component was uncommon. Each component, and each of the two subcomponents of extubation failure, was also analyzed separately using the same models and reported alongside the composite with equal emphasis. Because tracheostomy is a clinical decision influenced by physician judgment, institutional practice and family preference rather than a physiological event, a composite excluding tracheostomy is also reported (Supplementary Information S2, Panel A).

Secondary outcomes included ICU mortality, time to mortality, ICU length of stay, MV duration, and ventilator-free days at day 30.

### 2.6. Statistical Analysis

Continuous variables are presented as median (interquartile range) and compared by the Mann–Whitney U test and categorical variables as number (percentage) and compared by the chi-square or Fisher’s exact test. Analyses were stratified a priori by age group (younger <75 years; older ≥75 years). The 75-year threshold was pre-specified from the conventional ‘young-old’/’old-old’ distinction, the acceleration of frailty and sarcopenia beyond this age [13,20,21,22], and the cohort median age of 77 years; the limitations of dichotomizing age are addressed in the sensitivity analyses below. Because a scan obtained during the ICU stay could reflect rather than precede critical-illness catabolism, the primary analysis was also repeated after restricting the cohort to patients whose chest CT was obtained within ±7 days of ICU admission (Supplementary Information S1, Panels A and C). Multivariable logistic regression fitted by maximum likelihood was adjusted for a priori covariates: age, sex, BMI, APACHE II score, PaO_2_/FiO_2_ ratio, GCS score, vasopressor use, diabetes mellitus, chronic obstructive pulmonary disease (COPD), chronic heart failure and chronic kidney disease. Effect modification by age was tested in a pooled model containing a low thoracic muscle area × age group interaction term, evaluated by likelihood-ratio test, and its robustness examined by additional confounder adjustment, alternative modeling of age (continuous, quadratic, and cut-offs at 65, 70 and 80 years), and alternative exposure and outcome definitions (Supplementary Information S1 and S2).

Subgroup analyses examined the consistency of the association across six pre-specified variables: sex, APACHE II severity (<20 vs. ≥20), PaO_2_/FiO_2_ ratio category (<100, 100–200, ≥200), vasopressor use, corticosteroid use and diabetes mellitus). Effect modification was tested for each by a likelihood-ratio interaction test using the primary covariate set with the subgroup-defining variable omitted; these analyses were exploratory.

Data completeness was audited for every extracted variable (Supplementary Information S3). BMI (11.2%) and height (11.0%) were the only variables with more than 1% missing data; all other model covariates were complete. The primary analysis used complete cases, with multiple imputation for BMI as a sensitivity analysis (Supplementary Information S1). Discrimination (C-statistic), calibration (Hosmer–Lemeshow test, calibration slope, calibration-in-the-large and Brier score) and collinearity (variance inflation factors) were assessed, with optimism estimated by bootstrap internal validation using 300 resamples (Supplementary Information S4). Because multiple interaction tests were performed, *p* values within each pre-specified family were additionally adjusted by the Benjamini–Hochberg procedure (Supplementary Information S5); unadjusted values are quoted in the text and these analyses are regarded as exploratory.

Dose–response was assessed by categorizing thoracic muscle area into sex-specific quartiles derived from the 778-patient CT cohort, with the highest quartile as reference; linear trend was tested by entering quartile rank as an ordinal variable, and the association was also quantified per one-standard-deviation decrease in sex-standardized muscle area modeled continuously. A landmark analysis at day 7 examined whether the relationship with ICU mortality differed between deaths within 7 days and deaths thereafter among day-7 survivors.

Kaplan–Meier curves displayed time to death within 60 days by exposure status within each age group, compared by log-rank test. Because discharge alive from the ICU is unlikely to be non-informative for subsequent death, ICU mortality was additionally analyzed in a competing-risk framework treating ICU discharge as a competing event, using Aalen–Johansen cumulative incidence functions and Fine–Gray subdistribution hazard models (Supplementary Information S6 and S7). Analyses used R 4.5.1 (R Foundation for Statistical Computing, Vienna, Austria); two-tailed *p* < 0.05 was considered significant.

Effect modification by each subgroup variable was tested by a likelihood-ratio test in a single pooled model in the full cohort containing low thoracic muscle area, the subgroup variable, their product term, age group and the primary covariates. Stratum-specific estimates within each subgroup were obtained from models using the primary covariate set with the subgroup-defining variable omitted, because it is constant within that subgroup. These analyses were exploratory.

## 3. Results

### 3.1. Study Population and Baseline Characteristics

**Figure 1:**
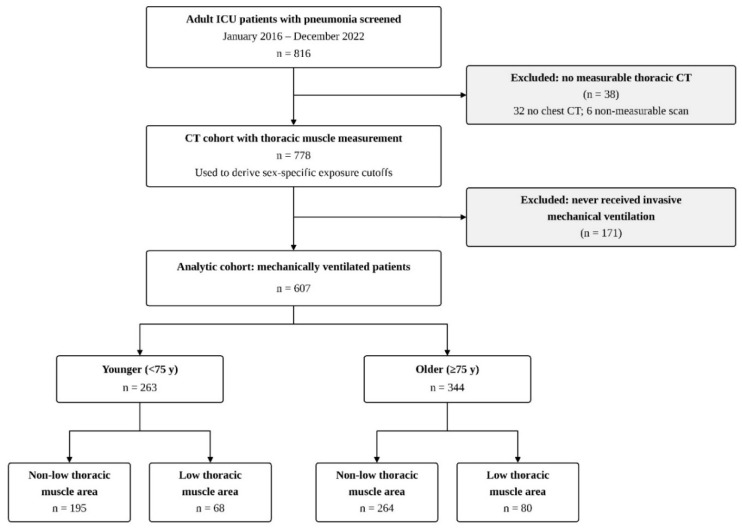
Flow diagram. Low thoracic muscle area = sex-specific lowest quartile (Q1) of the 778-patient CT cohort (male < 8729 mm^2^; female < 6715 mm^2^); non-low = quartiles 2 to 4.

Of 816 patients admitted to the ICU with pneumonia between January 2016 and December 2021, 38 were excluded because no measurable thoracic CT was available, leaving 778 patients with CT-based muscle measurement (Figure 1). Of these, 171 did not receive invasive MV and were excluded from the analysis of weaning outcomes, giving a final analytic cohort of 607 mechanically ventilated patients (78.0% of the CT cohort). The cohort was stratified into two age groups, 263 in the younger patients (age < 75 years) and 344 in the older patients (age ≥ 75 years). Within the mechanically ventilated cohort, 68 younger patients (25.9%) and 80 older patients (23.3%) were classified as having low thoracic muscle area based on sex-specific Q1 thresholds of thoracic muscle cross-sectional area (male: <8729 mm^2^; female: <6715 mm^2^).

Baseline characteristics are presented in Table 1. Low thoracic muscle area patients had markedly lower BMI than their counterparts without low thoracic muscle area in both age strata (median 15.8 vs. 21.2 kg/m^2^ in younger patients and 18.0 vs. 21.4 kg/m^2^ in older patients; both *p* < 0.001). This difference was large (standardized difference −1.29 in younger and −1.11 in older patients) and is discussed below as a potential source of confounding. The two exposure groups were not otherwise well balanced: 16 of 27 baseline variables in younger patients and 15 of 27 in older patients differed by a standardized difference exceeding 0.10 (Supplementary Information S8). Severity scores were similar in direction, with no significant differences in APACHE II, SOFA, SAPS-II, PaO_2_/FiO_2_ ratio, lactate, or vasopressor requirement (all *p* > 0.05). Younger patients with low thoracic muscle area had modestly lower systolic blood pressure (SBP) (median 110.5 vs. 123.0 mmHg, *p* = 0.019) and GCS score (median 9.0 vs. 11.0, *p* = 0.019) than their counterparts without low thoracic muscle area, and also lower creatinine (0.8 vs. 1.1 mg/dL, *p* < 0.001; standardized difference −0.43), lower hemoglobin (10.8 vs. 11.4 g/dL, *p* = 0.024), a lower prevalence of hypertension (25.0% vs. 40.5%, *p* = 0.022) and less frequent hemodialysis (8.8% vs. 19.5%, *p* = 0.042); APACHE II score was marginally higher (median 19.5 vs. 18.0, *p* = 0.050, standardized difference 0.33). These imbalances were not seen in the older stratum, whereas the principal categorical imbalance was a higher prevalence of cerebrovascular disease or dementia among patients with low thoracic muscle area (21.2% vs. 10.2%, *p* = 0.010). Major comorbidities, including diabetes mellitus, COPD, interstitial lung disease, chronic kidney disease, congestive heart failure, did not differ significantly in either stratum, with the exceptions of hypertension in younger patients and cerebrovascular disease or dementia in older patients noted above. Because these imbalances are neither few nor negligible, all multivariable models were adjusted for BMI, sex and comorbidity, and the possibility of residual confounding is addressed in Section 4.

Total thoracic muscle area was significantly lower in patients with low thoracic muscle area in both age groups (younger 7565.7 [6467.4–8014.1] vs. 10,963.6 [9450.9–12,460.2], *p* < 0.001; older 6788.0 [6142.2–8031.2] vs. 10249.3 [9160.8–11,573.5] mm^2^, *p* < 0.001).

### 3.2. Clinical Outcomes

Crude clinical outcomes are presented in Table 2. The pre-specified primary outcome of composite weaning failure occurred in 290 of 607 patients (47.8%) overall, comprising 129 of 263 younger patients (49.0%) and 161 of 344 older patients (46.8%). In younger patients, the incidence of composite weaning failure was substantially higher among patients with low thoracic muscle area than patients without low thoracic muscle area (69.1% [47/68] vs. 42.1% [82/195], *p* < 0.001), whereas in older patients the difference was smaller and did not reach statistical significance (55.0% [44/80] vs. 44.3% [117/264], *p* = 0.093). A similar age-dependent pattern was evident for prolonged MV (>21 days). The incidence was 2.6-fold higher among younger patients with low thoracic muscle area (14.7% vs. 5.6%, *p* = 0.018), whereas older patients with and without low thoracic muscle area did not differ (8.8% vs. 6.4%, *p* = 0.477).

In contrast, tracheostomy was more frequent among patients with low thoracic muscle area in both age strata to a similar degree (younger: 35.3% vs. 20.5%, *p* = 0.014; older: 30.0% vs. 17.4%, *p* = 0.014), so this component does not show the age-dependent pattern seen for the composite; after full adjustment the crude association in younger patients was no longer evident, while that in older patients persisted. Extubation failure did not differ significantly in either age stratum (younger: 45.6% vs. 33.3%, *p* = 0.071; older: 41.2% vs. 31.1%, *p* = 0.091). ICU mortality did not differ significantly between patients with and without low thoracic muscle area in either stratum (younger: 42.6% vs. 38.5%, *p* = 0.543; older: 50.0% vs. 42.8%, *p* = 0.256). ICU length of stay, MV duration, and ventilator-free days at day 30 did not differ significantly between patients with and without low thoracic muscle area within either stratum. Apart from the composite outcome and prolonged MV in younger patients, and tracheostomy in both strata, the crude outcome comparisons were therefore null, and we do not interpret the non-significant differences as supportive evidence.

### 3.3. ICU Survival According to Low Thoracic Muscle Area Status

**Figure 2:**
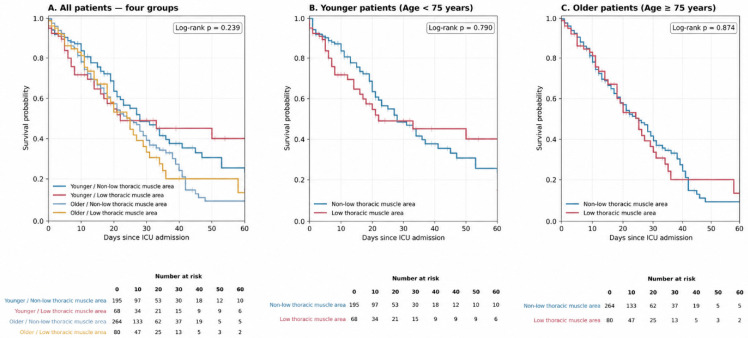
Kaplan–Meier survival curves according to low thoracic muscle area status. Curves were estimated by the Kaplan–Meier method and compared across groups by the log-rank test. Numbers at risk are shown below each panel. Short vertical tick marks on the curves indicate censoring events. (**A**): all four groups plotted together with the omnibus 4-group log-rank test (3 degrees of freedom). (**B**,**C**): within-age-stratum comparison of low thoracic muscle area vs. non-low thoracic muscle area patients. Within-stratum log-rank tests (low thoracic muscle area vs. non-low thoracic muscle area): Younger (**B**) *p* = 0.790; Older (**C**) *p* = 0.874. Abbreviations: ICU, intensive care unit; KM, Kaplan–Meier.

Kaplan–Meier survival analysis within each age stratum showed no significant overall difference (log-rank *p* = 0.239) in time-to-death between patients with and without low thoracic muscle area (Figure 2). Within the younger stratum, low thoracic muscle area and non-low thoracic muscle area patients had similar overall survival profiles (log-rank *p* = 0.790); because most patients were discharged alive rather than dying in the ICU, median survival times estimated from these curves are unstable and are not reported (Supplementary Information S9). Within the older patient stratum, survival curves were nearly superimposable (log-rank *p* = 0.874). These analyses are consistent with the multivariable finding that low thoracic muscle area was not independently associated with overall ICU mortality. In the younger patients, the survival curves separated during the first seven days. Seven-day survival was 75.9% in patients with low thoracic muscle area compared with 88.0% in patients without low thoracic muscle area (difference 12.1 percentage points). By 60 days, this gap reversed, with patients with low thoracic muscle area showing paradoxically higher estimated survival (40.2% vs. 23.1%), a pattern that would be expected if the early excess mortality depleted the higher-risk low-thoracic-muscle-area group, although this explanation is a post hoc interpretation and cannot be verified in these data. A post hoc landmark analysis at day 7 was performed to describe the timing of this pattern (Supplementary Information S10). Early mortality within 7 days of ICU admission was significantly higher among younger patients with low thoracic muscle area (20.6% vs. 10.8%, difference +9.8 percentage points, *p* = 0.040 by chi-square test and *p* = 0.060 by Fisher’s exact test), whereas mortality among day-7 survivors did not differ between patients with and without low thoracic muscle area (27.8% vs. 31.0%, *p* = 0.649). In the older patients, low thoracic muscle area was not associated with either early (13.8% vs. 11.7%, *p* = 0.631) or late mortality (42.0% vs. 35.2%, *p* = 0.301).

### 3.4. Association of Low Thoracic Muscle Area with Clinical Outcomes

**Figure 3:**
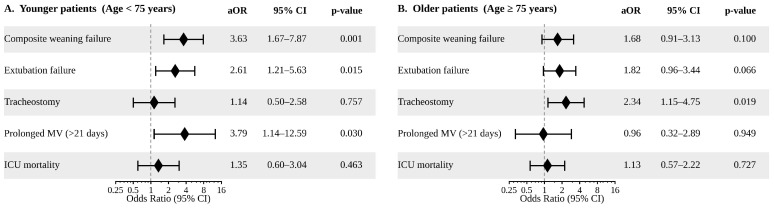
Forest plot of the association between low thoracic muscle area and clinical outcomes, stratified by age group. (**A**): Younger patients (Age < 75 years). (**B**): Older patients (Age ≥ 75 years). Interaction *p* (low-muscle-area × age group): composite weaning failure, 0.061; extubation failure, 0.436; tracheostomy, 0.805; prolonged MV (>21 days), 0.089; ICU mortality, 0.685. Multivariable models were adjusted for age, sex, body mass index, APACHE II score, PaO_2_/FiO_2_ ratio, Glasgow Coma Scale, vasopressor use, diabetes mellitus, chronic obstructive pulmonary disease, chronic heart failure and chronic kidney disease, and were fitted separately within each age stratum; the interaction *p*-value was obtained from a single model in the full cohort containing low thoracic muscle area, age group, their product term and the same covariates. Diamonds are point estimates and horizontal bars 95% CIs; the dashed line marks OR = 1, and the x-axis is on a log scale. Analyses were restricted to complete cases (*n* = 539; 233 younger, 306 older). Abbreviations: aOR, adjusted odds ratio; CI, confidence interval; APACHE II, Acute Physiology and Chronic Health Evaluation II; PaO_2_/FiO_2_, ratio of partial pressure of arterial oxygen to fraction of inspired oxygen; GCS, Glasgow Coma Scale; MV, mechanical ventilation; ICU, intensive care unit.

In fully adjusted models, low thoracic muscle area was associated with composite weaning failure in younger patients (aOR 3.63; 95% CI 1.67–7.87; *p* = 0.001) but not significantly in older patients (aOR 1.68; 95% CI 0.91–3.13; *p* = 0.100), and the age group interaction was not statistically significant (likelihood-ratio *p* = 0.061) (Figure 3, Supplementary Information S11). These analyses were restricted to complete cases (*n* = 539) because BMI was missing in 68 of 607 patients. Because the interaction test was not significant, these stratum-specific estimates should be read as showing an association that was present in both age groups with a larger estimate in younger patients, rather than as evidence of an association confined to younger patients.

Component-level estimates were less consistent. Extubation failure was associated with low thoracic muscle area more strongly in younger patients (aOR 2.61; 95% CI 1.21–5.63) than in older patients (1.82; 0.96–3.44; interaction *p* = 0.436), as was prolonged MV (3.79; 1.14–12.59 vs. 0.96; 0.32–2.89; interaction *p* = 0.089), although the latter rested on few events (20 and 23, respectively) and is imprecise. Tracheostomy showed the opposite pattern: after adjustment the association was confined to older patients (2.34; 1.15–4.75) and was no longer evident in younger patients (1.14; 0.50–2.58; interaction *p* = 0.805), in whom BMI rather than muscle area predicted the procedure (aOR 0.88 per kg/m^2^; 0.80–0.97; *p* = 0.008).

Low thoracic muscle area was not independently associated with ICU mortality in either age stratum after adjustment (younger aOR 1.35; 95% CI 0.60–3.04; older 1.13; 0.57–2.22; interaction *p* = 0.685).

The two subcomponents of extubation failure did not share a common age pattern: failure to achieve planned extubation was associated with low thoracic muscle area only in younger patients and reintubation within 48 h only in older patients, although neither subcomponent interaction reached significance (*p* = 0.114 and 0.217). Excluding tracheostomy, the association persisted in younger patients and remained non-significant in older patients (interaction *p* = 0.235). Counting the 36 patients who died within 48 h of an extubation not followed by reintubation as weaning failure, which increased the number of events from 290 to 322, likewise left the pattern unchanged (younger aOR 4.31, 95% CI 1.93–9.65; older 1.75, 0.92–3.31; interaction *p* = 0.040) (Supplementary Information S2, Panel C). Estimates were also stable to the derivation of the exposure threshold and to the use of the lowest quintile or tertile (Supplementary Information S2, Panel A).

In competing-risk analyses (Supplementary Information S6 and S7), low thoracic muscle area was not associated with the cumulative incidence of ICU death with ICU discharge as a competing event (younger sHR 1.42, 95% CI 0.81–2.48; older 1.07, 0.69–1.68; interaction *p* = 0.531). For liberation from MV, with death before liberation and tracheostomy as competing events, low muscle area was associated with a lower subdistribution hazard of liberation in younger (sHR 0.44, 0.26–0.76) but not older patients (0.73, 0.47–1.13; ratio of subdistribution hazard ratios 2.08, 1.09–3.94; *p* = 0.025).

### 3.5. Dose–Response Relationship Between Thoracic Muscle Mass and Composite Weaning Failure

**Figure 4:**
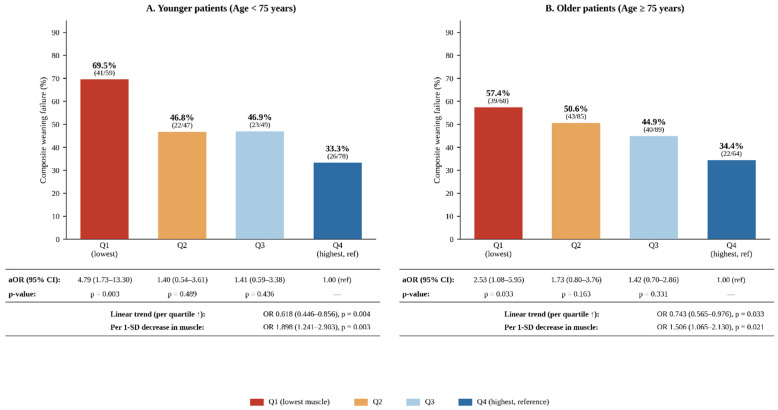
Dose–response analysis: muscle mass quartile and composite weaning failure stratified by age group. (**A**): Younger patients (Age < 75 years). (**B**): Older patients (Age ≥ 75 years). Adjusted odds ratios were estimated by multivariable logistic regression within each age stratum, using the same covariate set as the primary analysis: age, sex, body mass index, APACHE II score, PaO_2_/FiO_2_ ratio, Glasgow Coma Scale, vasopressor use, diabetes mellitus, chronic obstructive pulmonary disease, chronic heart failure and chronic kidney disease. Quartiles are sex-specific and were derived from the 778-patient CT cohort, with the highest quartile as the reference category. Two continuous analyses were performed using the same adjustment set. (1) Trend analysis, in which quartile rank was entered as a continuous variable taking values 1–4; an odds ratio below 1 indicates lower risk of weaning failure with higher muscle mass. (2) Per one-standard-deviation decrease in muscle mass, standardized within sex and with the sign reversed so that the odds ratio corresponds to a one-standard-deviation decrease. Analyses were restricted to complete cases because body mass index was missing in 68 of 607 patients. Alternative scalings of muscle mass, including muscle area indexed to height squared, are reported in Supplementary Information S12. Abbreviations: aOR, adjusted odds ratio; CI, confidence interval; SD, standard deviation; APACHE II, Acute Physiology and Chronic Health Evaluation II; PaO_2_/FiO_2_, ratio of partial pressure of arterial oxygen to fraction of inspired oxygen.

To evaluate whether the association reflected a continuous gradient rather than a binary threshold, thoracic muscle mass was analyzed across sex-specific quartiles within each age stratum, using the same fully adjusted models as the primary outcome analysis (Figure 4). In younger patients, composite weaning failure rates decreased progressively with increasing muscle mass (Q1 69.5%, Q2 46.8%, Q3 46.9%, Q4 33.3%); the Q1 vs. Q4 aOR was 4.79 (95% CI 1.73–13.30, *p* = 0.003), with a significant linear trend across quartiles (OR per quartile increase 0.618, 95% CI 0.446–0.856, *p* = 0.004). Modeled continuously, each one-standard-deviation decrease in thoracic muscle area was associated with a 1.90-fold increase in the odds of composite weaning failure (95% CI 1.24–2.90, *p* = 0.003). A gradient in the same direction was also present in older patients and likewise reached statistical significance (Q1 57.4%, Q2 50.6%, Q3 44.9%, Q4 34.4%; Q1 vs. Q4 aOR 2.53, 95% CI 1.08–5.95, *p* = 0.033; OR per quartile increase 0.743, 95% CI 0.565–0.976, *p* for trend 0.033; per one-standard-deviation decrease OR 1.51, 95% CI 1.06–2.13, *p* = 0.021).

Because absolute muscle area is not normalized to body size and is correlated with BMI, the analysis was repeated with muscle area indexed to height squared (Supplementary Information S12). On this scale the Q1 vs. Q4 contrast was no longer significant in younger patients (aOR 2.48, 95% CI 0.90–6.83, *p* = 0.080; per one-standard-deviation decrease OR 1.36, 95% CI 0.90–2.04, *p* = 0.140), although the test for trend remained significant (*p* = 0.042). In older patients the gradient remained significant on every measure (Q1 vs. Q4 aOR 3.12, 95% CI 1.28–7.63, *p* = 0.012; per one-standard-deviation decrease OR 1.47, 95% CI 1.04–2.08, *p* = 0.031; *p* for trend 0.012). The dose–response relationship is therefore not confined to younger patients, and the age contrast seen with absolute muscle area is substantially attributable to body-size heterogeneity that co-varies with age. These analyses are exploratory: the quartile models contain 14 parameters against 112 and 144 outcome events in the two strata, so individual quartile estimates are imprecise.

### 3.6. Sensitivity Analyses

Sensitivity analyses were performed to assess the robustness of the age-related interaction (Supplementary Information S1). It was not significant in the fully adjusted model (*p* = 0.061), and weakened further when corticosteroid exposure, the SOFA score or a comorbidity count was added (*p* = 0.073 to 0.133). Multiple imputation for missing BMI gave concordant results (*p* = 0.056). Height-normalizing muscle mass preserved the association in younger patients (aOR 2.43, 95% CI 1.16–5.10) but not in older patients (1.80, 0.93–3.48), and removed the interaction (*p* = 0.258). With age modeled continuously the adjusted odds ratio declined smoothly from 3.27 (95% CI 1.44–7.40) at 55 years to 1.83 (95% CI 1.03–3.24) at 85 years, remaining significant throughout, with no significant interaction (*p* = 0.250) and no departure from linearity (*p* = 0.235); alternative cut-offs at 65, 70 and 80 years gave interaction *p* = 0.402, 0.524 and 0.072. Restricting the cohort to the 521 patients (85.8%) whose chest CT was obtained within ±7 days of ICU admission, and thereby excluding the 51 patients (8.4%) scanned more than 7 days after admission, left the estimates unchanged (younger aOR 3.91, 95% CI 1.67–9.17; older 1.45, 0.73–2.89; *n* = 459); the interaction was nominally significant in this smaller subset (*p* = 0.027) but rests on fewer patients and events than the primary analysis, and our interpretation is based on the full cohort. Across every specification the direction of the association was preserved, with larger adjusted odds in younger patients, but the formal test of effect modification reached significance principally in models that did not adjust for BMI.

### 3.7. Subgroup Analyses

The association between low thoracic muscle area and composite weaning failure was examined across six pre-specified clinical subgroups using the same covariate set as the primary analysis (Table 3). The stronger effect in younger than in older patients was preserved in direction across all six subgroups (sex, APACHE II severity, PaO_2_/FiO_2_ category, vasopressor use, corticosteroid use and diabetes mellitus), with no evidence of effect modification in any (all interaction *p* ≥ 0.49; all Benjamini–Hochberg-adjusted q = 0.935).

## 4. Discussion

In this single-center retrospective cohort of 607 mechanically ventilated ICU patients with pneumonia, low CT-derived thoracic muscle area on the chest CT obtained closest to ICU admission was associated with composite weaning failure in younger patients (aOR 3.63, 95% CI 1.67–7.87) and, with a smaller and non-significant estimate, in older patients (aOR 1.68, 95% CI 0.91–3.13); the formal age interaction did not reach significance after full adjustment (*p* = 0.061). Across quartiles of muscle mass, the risk of weaning failure rose progressively in both age strata. Low muscle area was not independently associated with ICU mortality in either stratum.

The difference between age strata should be read as a difference in the magnitude of an association rather than as demonstrated effect modification. The interaction reached conventional significance principally in models that did not adjust for BMI; it weakened further when corticosteroid exposure, the SOFA score or a comorbidity count was added (*p* = 0.073 to 0.133), was removed by height-normalization of muscle area, and did not survive correction for multiple comparisons (Supplementary Information S1). With age modeled continuously the adjusted odds ratio declined smoothly from 3.27 at 55 years to 1.83 at 85 years and remained significant throughout, so the dichotomy at 75 years is a presentational convenience rather than a biological boundary. No individual component of the composite supported age modification independently (interaction *p* = 0.436 for extubation failure, 0.805 for tracheostomy, 0.089 for prolonged MV and 0.685 for ICU mortality). The contrast persisted in only one analysis, the competing-risk model for liberation from MV (ratio of subdistribution hazard ratios 2.08, 95% CI 1.09–3.94). These age-related observations should therefore be regarded as exploratory. The interpretation supported by these data is that low thoracic muscle area is associated with composite weaning failure across the age spectrum, and that whether its prognostic weight is genuinely larger in younger patients requires a prospective study designed and powered to test effect modification.

The more robust observation is the dose–response relationship. After full adjustment the odds of weaning failure increased across descending quartiles of muscle area in younger patients (lowest vs. highest quartile aOR 4.79, *p* for trend 0.004) and also in older patients (aOR 2.53, *p* for trend 0.033). When muscle area was indexed to height squared the gradient remained significant in older patients on every measure, while the quartile contrast in younger patients did not, indicating that the age contrast seen with absolute muscle area is substantially attributable to body-size heterogeneity that co-varies with age. Muscle mass therefore appears to carry prognostic information across the age range, and a categorical threshold is not required to describe the relationship.

Failure of ventilator liberation arises when respiratory pump demand exceeds capacity [3]. Thoracic muscle area reflects the global muscular envelope and correlates with diaphragmatic mass [23,24], and controlled ventilation produces rapid diaphragm atrophy [25] that predicts prolonged weaning, reintubation and tracheostomy [26,27]; low baseline reserve would plausibly be exhausted sooner by the same acute insult. The EWGSOP2 distinction between primary and secondary sarcopenia offers one account of why the association might be weaker in older patients, in whom low muscle mass would more often occur alongside declines in other physiological reserves [13]. We emphasize that this is a hypothesis and not a finding of the present study. We measured neither respiratory muscle strength, diaphragm thickness, frailty, nutritional status nor pre-ICU function, and cannot determine whether low muscle mass was primary or secondary in any individual patient. Three non-exclusive interpretations therefore remain open: a direct limitation of respiratory pump capacity, a marker of poor overall reserve, and a decision pathway in which visibly wasted patients prompt more conservative management. We accordingly describe the exposure as a marker associated with weaning failure rather than as a cause of it.

The composite was pre-specified as a pragmatic operational endpoint, not as a unified biological construct. Its components differ in severity and mechanism, and tracheostomy is a clinical decision influenced by physician judgment, institutional practice and family preference rather than a physiological event [28]. After full adjustment the association with tracheostomy was confined to older patients (aOR 2.34) and was no longer evident in younger patients (aOR 1.14), in whom BMI rather than muscle area predicted the procedure (aOR 0.88 per kg/m^2^). At the component level the association in younger patients was present both for extubation failure (aOR 2.61, 95% CI 1.21–5.63) and for prolonged MV (3.79, 1.14–12.59). Prolonged MV carried the larger point estimate but contributed only 20 of the 112 composite events in this stratum, with a correspondingly wide confidence interval, whereas extubation failure contributed 81; the composite-level signal cannot be attributed to any single component. The component-level estimates therefore carry equal or greater interpretive weight than the composite.

In contrast to previous reports [5,6,7,8,11], low muscle area was not independently associated with ICU mortality. Earlier meta-analyses pooled mixed surgical and medical cohorts with heterogeneous follow-up [5,7], whereas our outcome was confined to ICU death with discharge as a competing event [12]. A post hoc landmark analysis suggested that the null overall result in younger patients conceals a temporal pattern: mortality within the first 7 days was higher among those with low muscle area (20.6% vs. 10.8%, *p* = 0.040), with no further excess among day-7 survivors (27.8% vs. 31.0%, *p* = 0.649). This is a single exploratory comparison of borderline significance (Fisher’s exact *p* = 0.060) and requires confirmation.

No validated CT-based normative threshold exists for CT-derived thoracic muscle area, and the EWGSOP2 functional criteria cannot be measured in sedated, mechanically ventilated patients [10,16]. We therefore used sex-specific lowest-quartile cut-offs derived from the internal CT cohort, so that approximately one quarter of the cohort is exposed by construction and the threshold is not transferable to other populations. The exposure captures only the mass component of the sarcopenia construct, does not include the diaphragm, and was measured on a single scan, so long-standing loss cannot be distinguished from acute wasting and muscle quality is unknown. It may also act in part as a marker of severe underweight: median BMI among exposed younger patients was 15.8 kg/m^2^, below the World Health Organization threshold for severe thinness, and adjustment for BMI attenuated the association and removed the age interaction from statistical significance. We do not claim that the exposure identifies a muscle-loss phenotype independent of nutritional status. Thoracic muscle area was derived from a largely automated deep-learning segmentation of the chest CT rather than from manual tracing. This reduces, but does not eliminate, observer-dependent variability, because initial delineation on the workstation was radiologist-guided.

The segmentation network was externally validated in an independent hospital dataset (mean Dice similarity coefficient 0.92 to 0.94) [17], but it was developed using chest CT scans from this same institutional cohort, so its performance here may be more favorable than in an independent setting, and it was not re-validated against a manual reference standard within the present cohort. Systematic measurement error therefore cannot be excluded; because segmentation was performed without access to outcome data, any such error is expected to be non-differential with respect to the outcome. This cohort also overlaps with a previous report from our group that used the same imaging pipeline to derive muscle-mass clusters [17]; the present study differs in its research question, exposure definition and outcome, and no analysis is duplicated.

This retrospective single-center study requires prospective multicenter validation, particularly because tracheostomy timing, ICU admission thresholds and ventilator-liberation practice followed local rather than protocolized criteria. Exclusion of patients with a do-not-resuscitate order may have preferentially removed the frailest older patients and attenuated the association in that stratum. Because eligibility required a measurable scan rather than a fixed time window, muscle area measured during the ICU stay may partly reflect acute catabolism, raising the possibility of reverse causation; the timing analyses reported above argue against this but cannot exclude it. Pre-ICU functional status, duration of illness before admission, rehabilitation, delirium and treatment-limitation decisions were unrecorded, so residual confounding remains particularly in older patients. Several component and subgroup estimates rest on few events and are imprecise, and deaths shortly after extubation may in some cases represent palliative rather than successful liberation.

CT-derived thoracic muscle measurement is feasible without additional radiation, since chest CT is already routine in severe pneumonia [9], and interventions intended to preserve muscle, such as early mobilization and in-bed cycling, already exist [29]. We tested no intervention and assessed neither readiness for mobilization, feasibility and adherence, nor whether the effect of such interventions is modified by muscle mass. These data therefore support neither a risk-stratification threshold nor the targeting of any age group, both of which would require prospective evaluation.

## 5. Conclusions

In mechanically ventilated ICU patients with pneumonia, low CT-derived thoracic muscle area at admission was associated with composite weaning failure. The estimate was larger in patients younger than 75 years than in older patients, but the formal age interaction was not significant in the fully adjusted model and was further attenuated by height-normalization of muscle area, by additional confounder adjustment and by correction for multiple comparisons; the difference between strata should therefore be read as a difference in magnitude rather than as demonstrated effect modification. The graded association across the range of muscle mass, present in both age strata after full adjustment, is consistent with a continuous rather than a categorical threshold relationship, although these observational data cannot establish causation or identify a mechanism. Whether CT-based thoracic muscle assessment can improve risk stratification or guide muscle-preservation strategies remains to be tested prospectively.

## Figures and Tables

**Table 1:** Baseline characteristics.

Characteristic	Younger Patients (Age < 75 Years)			Older Patients (Age ≥ 75 Years)			Overall *p*-Value
	Non-Low Thoracic Muscle Area (*n* = 195)	Low Thoracic Muscle Area (*n* = 68)	*p*-Value	Non-Low Thoracic Muscle Area (*n* = 264)	Low Thoracic Muscle Area (*n* = 80)	*p*-Value	
Demographics							
Age (years)	63.0 (55.0–70.0)	65.0 (56.8–70.2)	0.430	82.0 (78.0–86.0)	83.0 (79.0–87.2)	0.052	<0.001
Male sex, *n* (%)	152 (77.9)	57 (83.8)	0.302	182 (68.9)	50 (62.5)	0.282	0.005
BMI (kg/m^2^)	21.2 (18.4–24.5)	15.8 (13.9–18.2)	<0.001	21.4 (19.3–24.4)	18.0 (16.2–19.6)	<0.001	<0.001
Clinical Severity Scores							
APACHE II score	18.0 (13.0–23.0)	19.5 (15.0–24.5)	0.050	20.0 (15.0–25.0)	21.0 (17.0–24.2)	0.502	0.003
SOFA score	11.0 (9.0–13.0)	10.0 (9.0–11.5)	0.241	10.0 (9.0–12.0)	11.0 (9.0–13.0)	0.293	0.288
SAPS-II score	45.0 (36.5–52.0)	44.5 (37.0–52.2)	0.890	48.0 (41.0–57.0)	50.5 (45.8–57.0)	0.082	<0.001
Glasgow coma scale	11.0 (6.0–14.0)	9.0 (6.0–14.0)	0.019	11.0 (7.0–14.0)	10.0 (6.0–13.0)	0.162	0.053
Laboratory values							
Systolic BP (mmHg)	123.0 (102.0–145.5)	110.5 (94.0–127.5)	0.019	126.5 (104.0–148.0)	120.0 (104.0–138.0)	0.072	0.008
Respiratory rate (breaths/min)	23.0 (18.0–28.0)	24.5 (20.0–28.0)	0.196	22.0 (19.8–27.0)	20.5 (18.0–25.0)	0.089	0.014
Temperature (°C)	36.9 (36.2–37.6)	36.6 (36.2–37.1)	0.065	36.9 (36.2–37.6)	37.0 (36.3–37.5)	0.959	0.285
WBC (×10^3^/μL)	11.3 (8.1–16.6)	11.6 (7.3–18.6)	0.550	11.3 (7.1–15.0)	10.7 (8.2–15.5)	0.559	0.628
CRP (mg/L)	13.2 (5.1–22.3)	14.4 (7.3–21.0)	0.351	13.1 (5.5–22.0)	14.2 (6.6–24.4)	0.258	0.493
Lactate (mmol/L)	2.6 (1.7–5.1)	2.3 (1.6–5.0)	0.635	2.4 (1.7–3.8)	2.3 (1.8–3.9)	0.968	0.498
Creatinine (mg/dL)	1.1 (0.9–1.9)	0.8 (0.6–1.3)	<0.001	1.2 (0.8–2.1)	1.2 (0.7–1.8)	0.275	<0.001
Hemoglobin (g/dL)	11.4 (9.6–13.2)	10.8 (9.0–12.0)	0.024	11.0 (9.6–12.6)	10.6 (9.6–11.7)	0.116	0.018
PaO_2_/FiO_2_ ratio	118.0 (80.8–176.2)	138.6 (87.5–214.8)	0.110	114.2 (76.9–190.2)	109.8 (75.9–189.0)	0.772	0.446
Comorbidities							
Hypertension, *n* (%)	79 (40.5)	17 (25.0)	0.022	177 (67.0)	54 (67.5)	0.940	<0.001
Diabetes mellitus, *n* (%)	71 (36.4)	18 (26.5)	0.136	103 (39.0)	35 (43.8)	0.449	0.156
COPD, *n* (%)	10 (5.1)	3 (4.4)	1.000	23 (8.7)	8 (10.0)	0.725	0.277
Interstitial lung disease, *n* (%)	5 (2.6)	1 (1.5)	1.000	18 (6.8)	1 (1.2)	0.088	0.030
Chronic kidney disease, *n* (%)	17 (8.7)	5 (7.4)	0.726	50 (18.9)	11 (13.8)	0.287	0.006
Chronic liver disease, *n* (%)	20 (10.3)	6 (8.8)	0.733	8 (3.0)	2 (2.5)	1.000	0.004
Congestive heart failure, *n* (%)	3 (1.5)	0 (0.0)	0.571	11 (4.2)	5 (6.2)	0.543	0.062
CVA/Dementia, *n* (%)	5 (2.6)	1 (1.5)	1.000	27 (10.2)	17 (21.2)	0.010	<0.001
ICU Interventions							
High-flow nasal cannula, *n* (%)	41 (21.0)	15 (22.1)	0.858	70 (26.5)	19 (23.8)	0.621	0.571
Vasopressor use, *n* (%)	92 (47.2)	27 (39.7)	0.286	108 (40.9)	36 (45.0)	0.516	0.520
Hemodialysis, *n* (%)	38 (19.5)	6 (8.8)	0.042	39 (14.8)	10 (12.5)	0.610	0.146
ECMO, *n* (%)	5 (2.6)	1 (1.5)	1.000	3 (1.1)	0 (0.0)	1.000	0.394
CT-Based Muscle Measurements							
Total thoracic muscle area (mm^2^)	10,963.6 (9450.9–12,460.2)	7565.7 (6467.4–8014.1)	<0.001	10,249.3 (9160.8–11573.5)	6788.0 (6142.2–8031.2)	<0.001	<0.001

Values are median (interquartile range) for continuous variables and *n* (%) for categorical variables. Within-group *p*-values: Mann–Whitney U test for continuous variables; Pearson chi-square test (or Fisher’s exact test when any expected cell count <5) for categorical variables. Overall *p*-values: Kruskal–Wallis H test for continuous variables; Pearson chi-square test across all 4 groups for categorical variables. Low thoracic muscle area = sex-specific lowest quartile (Q1) of the 778-patient CT cohort (male <8729 mm^2^; female <6715 mm^2^); non-low = quartiles 2 to 4. Abbreviations: APACHE II, Acute Physiology and Chronic Health Evaluation II; SOFA, Sequential Organ Failure Assessment; SAPS-II, Simplified Acute Physiology Score II; BP, blood pressure; WBC, white blood cell count; CRP, C-reactive protein; PaO_2_/FiO_2_, partial pressure of arterial oxygen to fraction of inspired oxygen; COPD, chronic obstructive pulmonary disease; CVA, cerebrovascular accident; ICU, intensive care unit; ECMO, extracorporeal membrane oxygenation; CT, computed tomography.

**Table 2:** Clinical outcomes.

Outcome	Younger Patients (Age < 75 Years)			Older Patients (Age ≥ 75 Years)			Overall *p*-Value
	Non-Low Thoracic Muscle Area (*n* = 195)	Low Thoracic Muscle Area (*n* = 68)	*p*-Value	Non-Low Thoracic Muscle Area (*n* = 264)	Low Thoracic Muscle Area (*n* = 80)	*p*-Value	
Primary Outcome							
Composite weaning failure, *n* (%)	82 (42.1)	47 (69.1)	<0.001	117 (44.3)	44 (55.0)	0.093	<0.001
Secondary Outcomes							
ICU mortality, *n* (%)	75 (38.5)	29 (42.6)	0.543	113 (42.8)	40 (50.0)	0.256	0.369
Time to mortality, days	19.0 (6.0–33.5)	8.0 (5.0–18.0)	0.042	13.0 (7.0–28.0)	16.5 (6.5–26.2)	0.926	0.188
ICU LOS, days	6.0 (2.0–15.0)	7.0 (4.0–18.5)	0.408	7.5 (3.0–14.0)	8.0 (3.0–15.2)	0.802	0.851
MV duration, days	5.0 (2.0–10.0)	6.0 (3.0–14.2)	0.061	6.0 (3.0–11.0)	6.0 (3.0–11.0)	0.594	0.076
Ventilator-free days at day 30	25.0 (20.0–28.0)	24.0 (15.8–27.0)	0.061	24.0 (19.0–27.0)	24.0 (19.0–27.0)	0.596	0.076
Extubation failure, *n* (%)	65 (33.3)	31 (45.6)	0.071	82 (31.1)	33 (41.2)	0.091	0.080
Never extubated	46 (23.6)	26 (38.2)	0.020	67 (25.4)	25 (31.2)	0.299	0.086
Reintubation, *n* (%)	19 (9.7)	5 (7.4)	0.556	15 (5.7)	8 (10.0)	0.176	0.355
Successful extubation, *n* (%)	130 (66.7)	37 (54.4)	0.071	182 (68.9)	47 (58.8)	0.091	0.080
Tracheostomy, *n* (%)	40 (20.5)	24 (35.3)	0.014	46 (17.4)	24 (30.0)	0.014	0.004
Prolonged MV (>21 days), *n* (%)	11 (5.6)	10 (14.7)	0.018	17 (6.4)	7 (8.8)	0.477	0.081
Organ Support							
Vasopressor use, *n* (%)	92 (47.2)	27 (39.7)	0.286	108 (40.9)	36 (45.0)	0.516	0.520
Hemodialysis, *n* (%)	38 (19.5)	6 (8.8)	0.042	39 (14.8)	10 (12.5)	0.610	0.146
ECMO, *n* (%)	5 (2.6)	1 (1.5)	1.000	3 (1.1)	0 (0.0)	1.000	0.394

Values are median (interquartile range) for continuous variables and *n* (%) for categorical variables. Within-group *p*-values: Mann–Whitney U test for continuous variables; Pearson chi-square test (or Fisher’s exact test when any expected cell count <5) for categorical variables. Overall *p*-values: Kruskal–Wallis H test for continuous variables; Pearson chi-square test across all 4 groups for categorical variables. Low thoracic muscle area = sex-specific lowest quartile (Q1) of the 778-patient CT cohort (male <8729 mm^2^; female <6715 mm^2^); non-low = quartiles 2 to 4.

**Table 3:** Subgroup analysis: Effect of low thoracic muscle area on weaning failure.

Subgroup	Younger Patients (Age < 75 Years)				Older Patients (Age ≥ 75 Years)				Interaction *p*
	Event Rate		Adjusted Model		Event Rate		Adjusted Model		
	Non-Low Thoracic Muscle Area (%)	Low Thoracic Muscle Area (%)	aOR (95% CI)	*p*	Non-Low Thoracic Muscle Area (%)	Low Thoracic Muscle Area (%)	aOR (95% CI)	*p*	
Overall	82/195 (42.1)	47/68 (69.1)	3.63 (1.67–7.87)	0.001	117/264 (44.3)	44/80 (55.0)	1.68 (0.91–3.13)	0.100	0.061
Sex									0.882
Male	62/152 (40.8)	39/57 (68.4)	3.62 (1.49–8.80)	0.004	82/182 (45.1)	25/50 (50.0)	1.43 (0.64–3.18)	0.379	
Female	20/43 (46.5)	8/11 (72.7)	1.46 (0.17–12.17)	0.727	35/82 (42.7)	19/30 (63.3)	1.99 (0.71–5.58)	0.193	
APACHE II severity									0.935
<20	46/113 (40.7)	24/34 (70.6)	4.08 (1.34–12.49)	0.014	54/125 (43.2)	18/37 (48.6)	1.88 (0.72–4.91)	0.198	
≥20	36/82 (43.9)	23/34 (67.6)	3.03 (0.93–9.84)	0.065	63/139 (45.3)	26/43 (60.5)	1.73 (0.73–4.11)	0.213	
PaO_2_/FiO_2_ ratio									0.497
<100	42/79 (53.2)	20/23 (87.0)	15.14 (2.41–95.16)	0.004	58/110 (52.7)	19/32 (59.4)	1.68 (0.61–4.61)	0.311	
100–200	27/81 (33.3)	17/25 (68.0)	3.38 (0.86–13.18)	0.080	39/97 (40.2)	15/28 (53.6)	1.74 (0.58–5.23)	0.322	
≥200	13/35 (37.1)	10/20 (50.0)	1.01 (0.16–6.57)	0.991	20/57 (35.1)	10/20 (50.0)	1.26 (0.30–5.30)	0.750	
Vasopressor use									0.635
Yes	53/92 (57.6)	21/27 (77.8)	1.56 (0.43–5.60)	0.495	53/108 (49.1)	23/36 (63.9)	2.82 (1.02–7.80)	0.047	
No	29/103 (28.2)	26/41 (63.4)	6.03 (2.07–17.53)	<0.001	64/156 (41.0)	21/44 (47.7)	1.37 (0.60–3.15)	0.454	
Corticosteroid use									0.605
Yes	62/128 (48.4)	38/49 (77.6)	4.79 (1.75–13.12)	0.002	89/187 (47.6)	27/52 (51.9)	1.43 (0.65–3.17)	0.372	
No	20/67 (29.9)	9/19 (47.4)	2.32 (0.55–9.91)	0.254	28/77 (36.4)	17/28 (60.7)	2.81 (0.95–8.32)	0.062	
Diabetes mellitus									0.829
Yes	29/71 (40.8)	13/18 (72.2)	3.68 (0.87–15.48)	0.076	40/103 (38.8)	18/35 (51.4)	1.32 (0.49–3.57)	0.583	
No	53/124 (42.7)	34/50 (68.0)	3.86 (1.48–10.08)	0.006	77/161 (47.8)	26/45 (57.8)	1.77 (0.78–4.01)	0.170	

Adjusted odds ratios were estimated separately in each subgroup and each age stratum by multivariable logistic regression using the same covariate set as the primary analysis: age, sex, body mass index, APACHE II score, PaO_2_/FiO_2_ ratio, Glasgow Coma Scale, vasopressor use, diabetes mellitus, chronic obstructive pulmonary disease, chronic heart failure and chronic kidney disease. The variable defining a subgroup was omitted from the model within that subgroup because it is constant there. In two strata a comorbidity term carried no information (no younger woman had chronic heart failure, and no younger patient with a PaO_2_/FiO_2_ ratio ≥200 had chronic obstructive pulmonary disease or chronic heart failure) and was likewise omitted from that cell only. Low thoracic muscle area = sex-specific lowest quartile (Q1) of the 778-patient CT cohort (male <8729 mm^2^; female <6715 mm^2^); non-low = quartiles 2 to 4. The interaction *p* value is the likelihood-ratio test *p* value for the low-muscle-area × subgroup interaction, obtained from a single model in the full cohort containing low muscle area, the subgroup variable, their product term, age group and the adjustment covariates; for the Overall row it is the low-muscle-area × age group interaction. After Benjamini–Hochberg adjustment across the six subgroup tests, no interaction remained significant (adjusted q = 0.935 for all six subgroup interactions: sex, APACHE II severity, PaO_2_/FiO_2_ ratio, vasopressor use, corticosteroid use and diabetes mellitus). Analyses were restricted to complete cases because body mass index was missing in 68 of 607 patients. Abbreviations: aOR, adjusted odds ratio; CI, confidence interval; APACHE II, Acute Physiology and Chronic Health Evaluation II.

## Data Availability

The data that support the findings of this study are available from the corresponding author upon reasonable request, subject to institutional data sharing agreements and applicable data privacy regulations.

## Supplementary Materials

The following supporting information can be downloaded at https://www.mdpi.com/article/10.3390/jcm15156105/s1. Supplementary Information S1: Sensitivity analyses of the association between low thoracic muscle area and composite weaning failure, by age stratum, and timing of the chest CT relative to ICU admission; Supplementary Information S2: Sensitivity of the primary result to outcome composition and to derivation of the exposure threshold; Supplementary Information S3: Completeness of extracted clinical and laboratory variables (analytic cohort, *n* = 607); Supplementary Information S4: Model diagnostics for the primary multivariable model: discrimination, calibration, and collinearity; Supplementary Information S5: Interaction *p* values before and after control for multiple comparisons (Benjamini–Hochberg); Supplementary Information S6: Competing-risk analysis of liberation from mechanical ventilation; Supplementary Information S7: Competing-risk analysis of ICU mortality with ICU discharge as the competing event; Supplementary Information S8: Baseline imbalance between patients with and without low thoracic muscle area: standardized differences; Supplementary Information S9: ICU all-cause mortality; Supplementary Information S10: Landmark Analysis: ICU Mortality by Early (≤7 days) vs Late (>7 days) Phase; Supplementary Information S11: Univariable and multivariable logistic regression analysis; Supplementary Information S12: Dose–response of composite weaning failure under alternative scalings of thoracic muscle mass.

## References

[1] Ferreira-Coimbra J., Sarda C., Rello J. (2020). Burden of Community-Acquired Pneumonia and Unmet Clinical Needs. Adv. Ther..

[2] (2024). GBD 2021 Lower Respiratory Infections and Antimicrobial Resistance Collaborators. Global, regional, and national incidence and mortality burden of non-COVID-19 lower respiratory infections and aetiologies, 1990–2021: A systematic analysis from the Global Burden of Disease Study 2021. Lancet Infect. Dis..

[3] Bureau C., Van Hollebeke M., Dres M. (2023). Managing respiratory muscle weakness during weaning from invasive ventilation. Eur. Respir. Rev..

[4] Pham T., Heunks L., Bellani G., Madotto F., Aragao I., Beduneau G., Goligher E.C., Grasselli G., Laake J.H., Mancebo J. (2023). Weaning from mechanical ventilation in intensive care units across 50 countries (WEAN SAFE): A multicentre, prospective, observational cohort study. Lancet Respir. Med..

[5] Jiang T., Lin T., Shu X., Song Q., Dai M., Zhao Y., Huang L., Tu X., Yue J. (2022). Prevalence and prognostic value of preexisting sarcopenia in patients with mechanical ventilation: A systematic review and meta-analysis. Crit. Care.

[6] Kou H.W., Yeh C.H., Tsai H.I., Hsu C.C., Hsieh Y.C., Chen W.T., Cheng H.T., Yu M.C., Lee C.W. (2019). Sarcopenia is an effective predictor of difficult-to-wean and mortality among critically ill surgical patients. PLoS ONE.

[7] Zhang X.M., Chen D., Xie X.H., Zhang J.E., Zeng Y., Cheng A.S. (2021). Sarcopenia as a predictor of mortality among the critically ill in an intensive care unit: A systematic review and meta-analysis. BMC Geriatr..

[8] Weijs P.J., Looijaard W.G., Dekker I.M., Stapel S.N., Girbes A.R., Oudemans-van Straaten H.M., Beishuizen A. (2014). Low skeletal muscle area is a risk factor for mortality in mechanically ventilated critically ill patients. Crit. Care.

[9] Mourtzakis M., Prado C.M., Lieffers J.R., Reiman T., McCargar L.J., Baracos V.E. (2008). A practical and precise approach to quantification of body composition in cancer patients using computed tomography images acquired during routine care. Appl. Physiol. Nutr. Metab..

[10] Moon S.W., Lee S.H., Woo A., Leem A.Y., Lee S.H., Chung K.S., Kim E.Y., Jung J.Y., Kang Y.A., Park M.S. (2022). Reference values of skeletal muscle area for diagnosis of sarcopenia using chest computed tomography in Asian general population. J. Cachexia Sarcopenia Muscle.

[11] Looijaard W.G., Dekker I.M., Stapel S.N., Girbes A.R., Twisk J.W., Oudemans-van Straaten H.M., Weijs P.J. (2016). Skeletal muscle quality as assessed by CT-derived skeletal muscle density is associated with 6-month mortality in mechanically ventilated critically ill patients. Crit. Care.

[12] Moisey L.L., Mourtzakis M., Cotton B.A., Premji T., Heyland D.K., Wade C.E., Bulger E., Kozar R.A. (2013). Skeletal muscle predicts ventilator-free days, ICU-free days, and mortality in elderly ICU patients. Crit. Care.

[13] Cruz-Jentoft A.J., Bahat G., Bauer J., Boirie Y., Bruyère O., Cederholm T., Cooper C., Landi F., Rolland Y., Sayer A.A. (2019). Sarcopenia: Revised European consensus on definition and diagnosis. Age Ageing.

[14] Laffey C.M., Sheerin R., Khazaei O., McNicholas B.A., Pham T., Heunks L., Bellani G., Brochard L., Tomescu D., Simpkin A.J. (2025). Impact of frailty and older age on weaning from invasive ventilation: A secondary analysis of the WEAN SAFE study. Ann. Intensive Care.

[15] Guidet B., de Lange D.W., Boumendil A., Leaver S., Watson X., Boulanger C., Szczeklik W., Artigas A., Morandi A., Andersen F. (2020). The contribution of frailty, cognition, activity of daily life and comorbidities on outcome in acutely admitted patients over 80 years in European ICUs: The VIP2 study. Intensive Care Med..

[16] Flaatten H., De Lange D.W., Morandi A., Andersen F.H., Artigas A., Bertolini G., Boumendil A., Cecconi M., Christensen S., Faraldi L. (2017). The impact of frailty on ICU and 30-day mortality and the level of care in very elderly patients (≥80 years). Intensive Care Med..

[17] Choi Y.H., Kim D.H., Jeon E.T., Lee H.J., Park T.Y., Yoon S.H., Jin K.N., Lee H.W. (2024). Cluster analysis of thoracic muscle mass using artificial intelligence in severe pneumonia. Sci. Rep..

[18] Hong J.H., Hong H., Choi Y.R., Kim D.H., Kim J.Y., Yoon J.-H., Yoon S.H. (2023). CT analysis of thoracolumbar body composition for estimating whole-body composition. Insights Imaging.

[19] Kim D.W., Ha J., Ko Y., Kim K.W., Park T., Lee J., You M.W., Yoon K.H., Park J.Y., Kee Y.J. (2021). Reliability of Skeletal Muscle Area Measurement on CT with Different Parameters: A Phantom Study. Korean J. Radiol..

[20] Collard R.M., Boter H., Schoevers R.A., Oude Voshaar R.C. (2012). Prevalence of frailty in community-dwelling older persons: A systematic review. J. Am. Geriatr. Soc..

[21] Berger M.J., Doherty T.J. (2010). Sarcopenia: Prevalence, mechanisms, and functional consequences. Interdiscip. Top. Gerontol..

[22] Fried L.P., Tangen C.M., Walston J., Newman A.B., Hirsch C., Gottdiener J., Seeman T., Tracy R., Kop W.J., Burke G. (2001). Frailty in older adults: Evidence for a phenotype. J. Gerontol. A Biol. Sci. Med. Sci..

[23] Peñuelas O., Keough E., López-Rodríguez L., Carriedo D., Gonçalves G., Barreiro E., Lorente J. (2019). Ventilator-induced diaphragm dysfunction: Translational mechanisms lead to therapeutical alternatives in the critically ill. Intensive Care Med. Exp..

[24] Berger D., Bloechlinger S., von Haehling S., Doehner W., Takala J., Z’Graggen W.J., Schefold J.C. (2016). Dysfunction of respiratory muscles in critically ill patients on the intensive care unit. J. Cachexia Sarcopenia Muscle.

[25] Levine S., Nguyen T., Taylor N., Friscia M.E., Budak M.T., Rothenberg P., Zhu J., Sachdeva R., Sonnad S., Kaiser L.R. (2008). Rapid disuse atrophy of diaphragm fibers in mechanically ventilated humans. N. Engl. J. Med..

[26] Goligher E.C., Dres M., Fan E., Rubenfeld G.D., Scales D.C., Herridge M.S., Vorona S., Sklar M.C., Rittayamai N., Lanys A. (2018). Mechanical Ventilation-induced Diaphragm Atrophy Strongly Impacts Clinical Outcomes. Am. J. Respir. Crit. Care Med..

[27] Dres M., Dubé B.P., Mayaux J., Delemazure J., Reuter D., Brochard L., Similowski T., Demoule A. (2017). Coexistence and Impact of Limb Muscle and Diaphragm Weakness at Time of Liberation from Mechanical Ventilation in Medical Intensive Care Unit Patients. Am. J. Respir. Crit. Care Med..

[28] Young D., Harrison D.A., Cuthbertson B.H., Rowan K. (2013). Effect of early vs late tracheostomy placement on survival in patients receiving mechanical ventilation: The TracMan randomized trial. JAMA.

[29] Schweickert W.D., Pohlman M.C., Pohlman A.S., Nigos C., Pawlik A.J., Esbrook C.L., Spears L., Miller M., Franczyk M., Deprizio D. (2009). Early physical and occupational therapy in mechanically ventilated, critically ill patients: A randomised controlled trial. Lancet.

