# Supplementary material for: Low CT-Derived Thoracic Muscle Area and Composite Weaning Failure in Mechanically Ventilated ICU Patients with Pneumonia: An Exploratory Analysis by Age Group
Source: J Clin Med. 2026 Aug 5;15(15):6105. doi: 10.3390/jcm15156105 (PMC13466258; doi:10.3390/jcm15156105)
Supplement: Supplementary file 1 [file jcm-15-06105-s001.zip › jcm-4432122-supplementary.pdf]

Low CT-derived thoracic muscle area and composite weaning failure in mechanically ventilated ICU patients with pneumonia: an exploratory analysis by age group

Supplementary Information

Supplementary information S1. Sensitivity analyses of the association between low thoracic muscle area and composite weaning failure, by age stratum, and timing of the chest CT relative to ICU admission

Supplementary information S2. Sensitivity of the primary result to outcome composition and to derivation of the exposure threshold

Supplementary information S3. Completeness of extracted clinical and laboratory variables (analytic cohort, n = 607)

Supplementary information S4. Model diagnostics for the primary multivariable model: discrimination, calibration, and collinearity

Supplementary information S5. Interaction P values before and after control for multiple comparisons (Benjamini–Hochberg)

Supplementary information S6. Competing-risk analysis of liberation from mechanical ventilation

Supplementary information S7. Competing-risk analysis of ICU mortality with ICU discharge as the competing event

Supplementary information S8. Baseline imbalance between patients with and without low thoracic muscle area: standardized differences

Supplementary information S9. ICU all cause mortality

Supplementary information S10. Landmark Analysis: ICU Mortality by Early (≤7 days)

vs Late (>7 days) Phase

Supplementary information S11. Univariable and multivariable logistic regression analysis

Supplementary information S12. Dose–response of composite weaning failure under alternative scalings of thoracic muscle mass

Supplementary information S1. Sensitivity analyses of the association between low thoracic muscle area and composite weaning failure, by age stratum, and timing of the chest CT relative to ICU admission

Panel A. Sensitivity analysis

	Younger (<75 y) aOR (95% CI)	Older (≥75 y) aOR (95% CI)	Interaction P	N
**Confounder adjustment**				
Age, sex, BMI, APACHE II, PaO_2/FiO_2, GCS, vasopressor, DM, COPD, CHF, CKD (primary)	3.63 (1.67–7.87)	1.68 (0.91–3.13)	0.061	539
+ corticosteroid exposure	3.62 (1.65–7.97)	1.68 (0.90–3.12)	0.088	539
+ SOFA score	3.47 (1.59–7.56)	1.70 (0.91–3.17)	0.089	539
+ comorbidity count in place of individual comorbidities	3.65 (1.69–7.89)	1.70 (0.92–3.16)	0.073	539
+ corticosteroid exposure and SOFA score	3.41 (1.54–7.54)	1.70 (0.91–3.17)	0.133	539
Multiple imputation for BMI	3.21 (1.59–6.45)	1.41 (0.82–2.43)	0.056	606
**Alternative exposure definition**				
Height-normalized index (area/$height^2$)[a]	2.43 (1.16–5.10)	1.80 (0.93–3.48)	0.258	539
Cut-offs derived from the ventilated cohort[b]	3.47 (1.61–7.46)	1.67 (0.91–3.08)	0.071	539
Continuous, per 1-SD decrease in muscle area	1.90 (1.24–2.90)	1.51 (1.06–2.13)	0.217	539
**Alternative age modelling**				
age as a continuous variable	—	—	0.250	539
age cutoff 65 years	2.83 (0.90–8.86)	2.08 (1.23–3.52)	0.402	539
age cutoff 70 years	2.64 (1.05–6.59)	2.05 (1.17–3.59)	0.524	539
age cutoff 75 years (primary)	3.63 (1.67–7.87)	1.68 (0.91–3.13)	0.061	539
age cutoff 80 years	3.24 (1.68–6.24)	1.46 (0.71–3.02)	0.072	539
**Alternative outcome / sample**				
Excluding ICU stay <72 hours	3.09 (1.35–7.08)	1.97 (0.98–3.96)	0.216	424
CT restricted to ±7 days of ICU admission	3.91 (1.67–9.17)	1.45 (0.73–2.89)	0.027	459

Panel B. Adjusted odds ratio by age, from the fully adjusted model with age entered continuously

Age (years)	Adjusted OR for low thoracic muscle area (95% CI)
55	3.27 (1.44–7.40)
65	2.69 (1.51–4.80)
75	2.22 (1.39–3.55)
85	1.83 (1.03–3.24)
Test for departure from a linear age effect, P	0.235

Panel C. Timing of the chest CT used for muscle measurement relative to ICU admission

Timing of chest CT relative to ICU admission	Overall (n = 607)	Non-low thoracic muscle area (n = 459)	Low thoracic muscle area (n = 148)	P
**Distribution of CT timing, n (%)**				**0.223**
More than 7 days before ICU admission	35 (5.8)	26 (5.7)	9 (6.1)	
1–7 days before ICU admission	229 (37.7)	165 (35.9)	64 (43.2)	
Day of ICU admission	227 (37.4)	174 (37.9)	53 (35.8)	

Timing of chest CT relative to ICU admission	Overall (n = 607)	Non-low thoracic muscle area (n = 459)	Low thoracic muscle area (n = 148)	P
1–7 days after ICU admission	65 (10.7)	56 (12.2)	9 (6.1)	
More than 7 days after ICU admission	51 (8.4)	38 (8.3)	13 (8.8)	
**Grouped summaries, n (%)**				
On or before the day of ICU admission	491 (80.9)	365 (79.5)	126 (85.1)	0.164
After ICU admission	116 (19.1)	94 (20.5)	22 (14.9)	0.164
Within ±7 days of ICU admission	521 (85.8)	395 (86.1)	126 (85.1)	0.885
**Interval between CT and ICU admission, days**				
Median (IQR)	0 (−1 to 0)	0 (−1 to 0)	0 (−2 to 0)	0.078
Full range	−33 to +363	−33 to +363	−31 to +30	—

Panel A. All models are multivariable logistic regression models fitted separately within each age stratum, adjusted for age, sex, body mass index, APACHE II score, PaO2/FiO2 ratio, Glasgow Coma Scale, vasopressor use, diabetes mellitus, chronic obstructive pulmonary disease, chronic heart failure and chronic kidney disease unless otherwise stated. Interaction P values are from likelihood-ratio tests comparing pooled models with and without the exposure-by-age-group product term. N denotes the pooled analytic sample for the interaction test.The exposure is low thoracic muscle area, defined as the sex-specific lowest quartile in the 778-patient CT cohort (male <8,729 mm2; female <6,715 mm2) unless otherwise stated. [a]Muscle area indexed to height squared (male <3,164; female <2,901 mm2/m2). [b]Cut-offs derived from the 607 ventilated patients (male <8,844 mm2; female <6,721 mm2).

Abbreviations: aOR, adjusted odds ratio; CI, confidence interval; APACHE II, Acute Physiology and Chronic Health Evaluation II; BMI, body mass index; DM, diabetes mellitus; COPD, chronic obstructive pulmonary disease; CHF, chronic heart failure; CKD, chronic kidney disease; SD, standard deviation.

Panel B. The odds ratio declines smoothly with advancing age and remains significant across the observed range, and a quadratic term provides no evidence of departure from a linear age effect. These analyses give no statistical support for a discrete threshold at 75 years or at any other age, and the dichotomy used in the main analysis should therefore be regarded as a presentational convenience. Stratum-specific estimates are stable across all covariate sets in Panel A, whereas the age interaction weakens as further confounders are added.

Panel C. Negative values indicate that the chest CT preceded ICU admission. Eligibility required a measurable scan rather than a fixed time window. Percentages are calculated within each column. P values are from the chi-square test for the categorical comparisons between exposure groups and from the Mann–Whitney U test for the median interval. CT timing did not differ appreciably between exposure groups, and in most patients the scan was obtained on or before the day of ICU admission rather than during the ICU stay. The corresponding sensitivity analysis restricted to scans obtained within ±7 days is shown in Panel A.

Supplementary information S2. Sensitivity of the primary result to outcome composition and to derivation of the exposure threshold

Panel A. Composition of the composite outcome

Outcome or exposure definition	Younger, aOR (95% CI)	Older, aOR (95% CI)	Interaction P
Composite as defined (primary)	3.63 (1.67–7.87), P=0.001	1.68 (0.91–3.13), P=0.100	0.061
Composite excluding tracheostomy	2.99 (1.38–6.47), P=0.005	1.56 (0.83–2.91), P=0.166	0.235
Extubation failure (both subcomponents)	2.61 (1.21–5.63), P=0.015	1.82 (0.96–3.44), P=0.066	0.436
Failure to achieve planned extubation	2.94 (1.27–6.81), P=0.012	1.31 (0.66–2.58), P=0.439	0.114
Reintubation within 48 h of planned extubation	1.10 (0.28–4.23), P=0.893	3.23 (1.06–9.82), P=0.039	0.217
Tracheostomy alone	1.14 (0.50–2.58), P=0.757	2.34 (1.15–4.75), P=0.019	0.805
Prolonged mechanical ventilation alone	3.79 (1.14–12.59), P=0.030	0.96 (0.32–2.89), P=0.949	0.089

Panel B. Derivation of the exposure threshold (composite outcome)

Outcome or exposure definition	Younger, aOR (95% CI)	Older, aOR (95% CI)	Interaction P
Lowest quartile, 778-patient CT cohort (primary)	3.63 (1.67–7.87), P=0.001	1.68 (0.91–3.13), P=0.100	0.061
Lowest quartile, 607 ventilated patients	3.47 (1.61–7.46), P=0.001	1.67 (0.91–3.08), P=0.100	0.071
Lowest quintile, 778-patient CT cohort	3.33 (1.47–7.53), P=0.004	1.73 (0.89–3.36), P=0.106	0.139
Lowest tertile, 778-patient CT cohort	2.24 (1.09–4.61), P=0.029	1.70 (0.95–3.02), P=0.072	0.246

Panel C. Composition of the composite weaning-failure outcome and incorporation of death (analytic cohort, n = 607)

Classification	n	% of 607
**Combination of components met (mutually exclusive)**		
Extubation failure only	137	22.6
Tracheostomy only	56	9.2
Prolonged mechanical ventilation only	10	1.6
Extubation failure and tracheostomy	52	8.6
Extubation failure and prolonged mechanical ventilation	9	1.5
Tracheostomy and prolonged mechanical ventilation	13	2.1
All three components	13	2.1
No component (non-event)	317	52.2
**Composite weaning failure (any component)**	**290**	**47.8**
**Number of components met per patient with composite weaning failure**		
One component	203	33.4
Two components	74	12.2
Three components	13	2.1
**Component totals (patients may appear in more than one row)**		
Extubation failure	211	34.8
Failure to achieve planned extubation (never extubated)	164	27.0
Reintubation within 48 hours of planned extubation	47	7.7
Tracheostomy	134	22.1
Prolonged mechanical ventilation (>21 days)	45	7.4
**Sum of component occurrences**	**390**	–

Classification	n	% of 607
**Incorporation of death**		
ICU deaths	257	42.3
Deaths in patients meeting the composite	160	26.4
Deaths in patients not meeting the composite	97	16.0
Died without successful extubation	132	21.7
Never extubated	116	19.1
Reintubated within 48 hours, no subsequent successful extubation	16	2.6
Patients classified as composite events on the basis of death alone	0	0.0
Died within 48 hours of an extubation not followed by reintubation	36	5.9

Panel D. Sensitivity of the primary result to the handling of death shortly after extubation

Outcome definition	Younger (<75 y), aOR (95% CI)	Older (≥75 y), aOR (95% CI)	Interaction P
Composite as defined (primary); 290 events	3.63 (1.67–7.87)	1.68 (0.91–3.13)	0.061
Composite additionally counting death within 48 hours of an extubation not followed by reintubation; 322 events	4.31 (1.93–9.65)	1.75 (0.92–3.31)	0.040

Panel B. All models are adjusted for age, APACHE II score, PaO_2/FiO_2 ratio, Glasgow Coma Scale, vasopressor use, body mass index, sex, diabetes mellitus, chronic obstructive pulmonary disease, chronic heart failure and chronic kidney disease, and are fitted separately within each age stratum.

Panel C. 36 patients died within 48 hours of an extubation that was not followed by reintubation and were classified as having achieved extubation under the primary definition; some of these are likely to represent palliative extubation rather than genuine ventilator liberation. Counting them as weaning failure increased the number of events from 290 to 322 and did not change the pattern of association (lower table). Models are multivariable logistic regressions fitted separately within each age stratum and adjusted for age, sex, body mass index, APACHE II score, PaO_2/FiO_2 ratio, Glasgow Coma Scale, vasopressor use, diabetes mellitus, chronic obstructive pulmonary disease, chronic heart failure and chronic kidney disease, restricted to complete cases (n = 539; 233 younger, 306 older); interaction P values are from likelihood-ratio tests.

Supplementary information S3. Completeness of extracted clinical and laboratory variables (analytic cohort, n = 607)

Variable	Available, n/N	Missing, %
Age	607/607	0.0%
Sex	607/607	0.0%
Body mass index	539/607	11.2%
Height	540/607	11.0%
APACHE II score	607/607	0.0%
SOFA score	606/607	0.2%
SAPS-II score	607/607	0.0%
Systolic blood pressure	607/607	0.0%
Diastolic blood pressure	607/607	0.0%
Respiratory rate	607/607	0.0%
Body temperature	607/607	0.0%
PaO_2/FiO_2 ratio	607/607	0.0%
Arterial pH	607/607	0.0%
$PaCO_2$	607/607	0.0%
Bicarbonate	607/607	0.0%
White blood cell count	607/607	0.0%
Hemoglobin	607/607	0.0%
Platelet count	607/607	0.0%
C-reactive protein	607/607	0.0%
Creatinine	607/607	0.0%
Lactate	607/607	0.0%
Glasgow Coma Scale	607/607	0.0%
Hypertension	607/607	0.0%
Diabetes mellitus	607/607	0.0%
COPD	607/607	0.0%
Interstitial lung disease	607/607	0.0%
Chronic kidney disease	607/607	0.0%
Chronic liver disease	607/607	0.0%
Congestive heart failure	607/607	0.0%
Cerebrovascular disease / dementia	607/607	0.0%
Vasopressor use	607/607	0.0%
High-flow nasal cannula	607/607	0.0%
Hemodialysis	607/607	0.0%
ECMO	607/607	0.0%
MV duration	607/607	0.0%
Extubation	607/607	0.0%
Reintubation	606/607	0.2%
Tracheostomy	606/607	0.2%
Survival status	607/607	0.0%

Variable	Available, n/N	Missing, %
Thoracic muscle area	607/607	0.0%

Completeness refers to the 607 mechanically ventilated patients included in the analysis of weaning outcomes. Body mass index and height were the only variables with more than 1% missing data. The primary analysis used complete cases, and a multiple-imputation sensitivity analysis for body mass index is reported in Supplementary information 1.

Supplementary information S4. Model diagnostics for the primary multivariable model: discrimination, calibration, and collinearity

Panel A. Discrimination and calibration

Metric	Younger (<75 y)	Older (≥75 y)
Patients / outcome events	233 / 112	306 / 144
Events per candidate variable	9.3	12.0
C-statistic, apparent (95% CI)	0.746 (0.682–0.809)	0.656 (0.595–0.717)
C-statistic, optimism-corrected	0.695	0.593
Hosmer–Lemeshow χ^2 (P)	13.75 (0.089)	7.61 (0.473)
Calibration slope, apparent	1.00	1.00
Calibration slope, optimism-corrected	0.61	0.62
Calibration-in-the-large	-0.000	0.000
Brier score	0.204	0.232

Panel B. Collinearity (variance inflation factors, pooled model)

Covariate	VIF
Age (years)	2.88
Age group (≥75 y)	2.77
APACHE II score	1.83
Glasgow Coma Scale	1.73
Body mass index	1.31
Low thoracic muscle area	1.24
Chronic kidney disease	1.10
COPD	1.10
Diabetes mellitus	1.07
Male sex	1.07
Vasopressor use	1.05
PaO_2/FiO_2 ratio	1.05
Chronic heart failure	1.04

Optimism in the C-statistic and calibration slope was estimated by bootstrap internal validation with 300 resamples (Harrell's method). Calibration slopes below 1 after optimism correction indicate some overfitting, as expected for models of this size in a cohort of this size, and support the interpretation of these models as explanatory rather than as validated prediction tools. No variance inflation factor exceeded 3, indicating no material collinearity among covariates.

Supplementary information S5. Interaction P values before and after control for multiple comparisons (Benjamini–Hochberg)

Test	Unadjusted P	BH-adjusted q
**Family 1: age interaction across the five outcomes**		
Composite weaning failure	0.061	0.223
Extubation failure	0.436	0.727
Tracheostomy	0.805	0.805
Prolonged MV (>21 days)	0.089	0.223
ICU mortality	0.685	0.805
**Family 2: exposure × subgroup interaction (Table 3)**		
Sex	0.882	0.935
APACHE II severity	0.935	0.935
PaO_2/FiO_2 ratio	0.497	0.935
Vasopressor use	0.635	0.935
Corticosteroid use	0.605	0.935
Diabetes mellitus	0.829	0.935

The Benjamini–Hochberg procedure was applied separately within each pre-specified family of tests (five outcomes in Family 1; six subgroups in Family 2), with q values computed as p(i)×m/i over p values ranked in ascending order and enforced to be monotone non-decreasing. Family 2 P values are those reported in Table 3 of the main text. After adjustment, no age-by-outcome interaction reached conventional significance, and no subgroup interaction reached conventional significance either. The mechanical-ventilation initiation route subgroup, which was significant in an earlier version of this analysis, has been removed from Table 3 because its stratum-specific estimates were too imprecise to interpret.

Supplementary information S6. Competing-risk analysis of liberation from mechanical ventilation

	Younger patients (age < 75 y)		Older patients (age ≥ 75 y)	
	Non-low	Low	Non-low	Low
**Terminal ventilator outcome, n (%)**				
Successful liberation	115/195 (59.0)	22/68 (32.4)	153/264 (58.0)	37/80 (46.3)
Tracheostomy	40/195 (20.5)	24/68 (35.3)	46/264 (17.4)	24/80 (30.0)
Death without liberation	34/195 (17.4)	15/68 (22.1)	52/264 (19.7)	12/80 (15.0)
Still ventilated at censoring	6/195 (3.1)	7/68 (10.3)	13/264 (4.9)	7/80 (8.8)
**Cumulative incidence of successful liberation (Aalen–Johansen)**				
Day 14	55.7%	29.8%	52.4%	44.1%
Day 28	60.6%	31.4%	59.9%	50.2%
**Cumulative incidence of death before liberation**				
Day 14	15.3%	19.5%	15.2%	13.0%
Day 28	18.0%	22.8%	20.2%	14.5%
**Effect of low thoracic muscle area, adjusted**				
Liberation — Fine–Gray sHR (95% CI)	0.44 (0.26–0.76), P=0.003		0.73 (0.47–1.13), P=0.160	
Liberation — cause-specific HR	0.41 (0.24–0.71), P=0.001		0.81 (0.52–1.26), P=0.349	
Death before liberation — Fine–Gray sHR	2.26 (0.97–5.24), P=0.058		0.69 (0.32–1.48), P=0.336	
Death before liberation — cause-specific HR	1.55 (0.67–3.57), P=0.302		0.75 (0.33–1.68), P=0.486	
**Age interaction (ratio of sHRs)**	Liberation: 2.08 (1.09–3.94), P=0.025		Death: 0.42 (0.15–1.17), P=0.098	

Terminal ventilator outcomes and cumulative incidence estimates are based on all 607 patients. The four terminal ventilator outcomes are mutually exclusive and jointly account for all 607 patients: successful liberation (n=327), tracheostomy (n=134), death without liberation (n=113) and still ventilated at censoring (n=33). Successful liberation denotes extubation sustained for more than 48 hours without tracheostomy; death without liberation denotes death in a patient who never achieved sustained extubation and did not undergo tracheostomy. These states map onto the composite weaning-failure outcome of the main analysis as follows: the 113 patients who died without liberation had by definition not achieved planned extubation and are therefore already contained within the extubation-failure component of the composite, and patients who underwent tracheostomy are contained within the tracheostomy component; because tracheostomy is treated here as a separate terminal state, the 132 deaths without successful extubation in the main analysis are distributed between the death-without-liberation state (113) and the tracheostomy state (19). Patients meeting more than one component of the composite contribute a single composite event, and no patient was classified as a composite event on the basis of death alone (Supplementary information 2, Panel C).

Regression models were fitted separately within each age stratum and adjusted for age, APACHE II score, PaO_2/FiO_2 ratio, Glasgow Coma Scale, vasopressor use, body mass index, sex, diabetes mellitus, chronic obstructive pulmonary disease, chronic heart failure, and chronic kidney disease; they were restricted to complete cases (young n = 233, 124 liberation and 36 death events; elderly n = 306, 169 liberation and 55 death events).

Fine–Gray models estimate the effect on the cumulative incidence of the event (subdistribution hazard); cause-specific Cox models estimate the effect on the instantaneous rate among those still ventilated. The age interaction is the ratio of subdistribution hazard ratios (low muscle area × age group) from a pooled Fine–Gray model containing both strata.

Abbreviations: sHR, subdistribution hazard ratio; HR, hazard ratio; CI, confidence interval.

Supplementary information S7. Competing-risk analysis of ICU mortality with ICU discharge as the competing event

	Younger, non-low	Younger, low
Cumulative incidence of ICU death, day 28	36.9%	39.7%
Cumulative incidence of ICU death, day 60	38.5%	42.6%
Fine–Gray sHR (95% CI), younger	1.42 (0.81–2.48)	P = 0.216
	**Older, non-low**	**Older, low**
Cumulative incidence of ICU death, day 28	39.4%	45.0%
Cumulative incidence of ICU death, day 60	42.4%	50.0%
Fine–Gray sHR (95% CI), older	1.07 (0.69–1.68)	P = 0.761
Age interaction (ratio of sHRs)	0.81 (0.43–1.55)	P = 0.531

Time was measured from ICU admission. ICU death was the event of interest and discharge alive from the ICU was treated as a competing event rather than as non-informative censoring. Fine–Gray models were adjusted for age, sex, body mass index, APACHE II score, PaO_2/FiO_2 ratio, Glasgow Coma Scale, vasopressor use, diabetes mellitus, chronic obstructive pulmonary disease, chronic heart failure, and chronic kidney disease. Low thoracic muscle area was not associated with the cumulative incidence of ICU death in either age stratum, consistent with the Kaplan–Meier analysis but without relying on the assumption that ICU discharge is non-informative.

Supplementary information S8. Baseline imbalance between patients with and without low thoracic muscle area: standardized differences

Variable	Younger: non-low	Younger: low	SMD	Older: non-low	Older: low	SMD
Age, years *	63.0 (55.0–70.0)	65.0 (56.8–70.2)	0.03	82.0 (78.0–86.0)	83.0 (79.0–87.2)	0.23
Male sex *	152 (77.9)	57 (83.8)	0.15	182 (68.9)	50 (62.5)	-0.14
Body mass index, kg/m² *	21.2 (18.4–24.5)	15.8 (13.9–18.2)	-1.29	21.4 (19.3–24.4)	18.0 (16.2–19.6)	-1.11
APACHE II score *	18.0 (13.0–23.0)	19.5 (15.0–24.5)	0.33	20.0 (15.0–25.0)	21.0 (17.0–24.2)	0.08
SOFA score *	11.0 (9.0–13.0)	10.0 (9.0–11.5)	-0.14	10.0 (9.0–12.0)	11.0 (9.0–13.0)	0.13
SAPS-II score *	45.0 (36.5–52.0)	44.5 (37.0–52.2)	0.07	48.0 (41.0–57.0)	50.5 (45.8–57.0)	0.18
Glasgow Coma Scale *	11.0 (6.0–14.0)	9.0 (6.0–14.0)	-0.29	11.0 (7.0–14.0)	10.0 (6.0–13.0)	-0.17
Systolic blood pressure, mmHg *	123.0 (102.0–145.5)	110.5 (94.0–127.5)	-0.34	126.5 (104.0–148.0)	120.0 (104.0–138.0)	-0.30
Respiratory rate, /min *	23.0 (18.0–28.0)	24.5 (20.0–28.0)	0.23	22.0 (19.8–27.0)	20.5 (18.0–25.0)	-0.24
Temperature, °C *	36.9 (36.2–37.6)	36.6 (36.2–37.1)	-0.24	36.9 (36.2–37.6)	37.0 (36.3–37.5)	-0.03
PaO_2/FiO_2 ratio *	118.0 (80.8–176.2)	138.6 (87.5–214.8)	0.25	114.2 (76.9–190.2)	109.8 (75.9–189.0)	-0.03
White blood cells, $\times 10^3/\mu L$	11.3 (8.1–16.6)	11.6 (7.3–18.6)	0.09	11.3 (7.1–15.0)	10.7 (8.2–15.5)	0.10
C-reactive protein, mg/L *	13.2 (5.1–22.3)	14.4 (7.3–21.0)	0.08	13.1 (5.5–22.0)	14.2 (6.6–24.4)	0.16
Lactate, mmol/L	2.6 (1.7–5.1)	2.3 (1.6–5.0)	0.00	2.4 (1.7–3.8)	2.3 (1.8–3.9)	0.09
Creatinine, mg/dL *	1.1 (0.9–1.9)	0.8 (0.6–1.3)	-0.43	1.2 (0.8–2.1)	1.2 (0.7–1.8)	-0.12
Hemoglobin, g/dL *	11.4 (9.6–13.2)	10.8 (9.0–12.0)	-0.27	11.0 (9.6–12.6)	10.6 (9.6–11.7)	-0.21
Hypertension *	79 (40.5)	17 (25.0)	-0.34	177 (67.0)	54 (67.5)	0.01
Diabetes mellitus *	71 (36.4)	18 (26.5)	-0.22	103 (39.0)	35 (43.8)	0.10
COPD	10 (5.1)	3 (4.4)	-0.03	23 (8.7)	8 (10.0)	0.04
Interstitial lung disease *	5 (2.6)	1 (1.5)	-0.08	18 (6.8)	1 (1.2)	-0.29
Chronic kidney disease *	17 (8.7)	5 (7.4)	-0.05	50 (18.9)	11 (13.8)	-0.14
Chronic liver disease	20 (10.3)	6 (8.8)	-0.05	8 (3.0)	2 (2.5)	-0.03
Congestive heart failure *	3 (1.5)	0 (0.0)	-0.18	11 (4.2)	5 (6.2)	0.09
Cerebrovascular disease / dementia *	5 (2.6)	1 (1.5)	-0.08	27 (10.2)	17 (21.2)	0.31
Vasopressor use *	92 (47.2)	27 (39.7)	-0.15	108 (40.9)	36 (45.0)	0.08
Hemodialysis *	38 (19.5)	6 (8.8)	-0.31	39 (14.8)	10 (12.5)	-0.07
ECMO *	5 (2.6)	1 (1.5)	-0.08	3 (1.1)	0 (0.0)	-0.15

Standardized differences are reported because they do not depend on sample size; an absolute value above 0.10 is conventionally taken to indicate meaningful imbalance. Continuous variables are median (interquartile range) and categorical variables n (%).

* $|SMD| > 0.10$ in at least one age stratum. 16 of 27 baseline variables in younger patients and 15 of 27 in older patients exceeded this threshold, so the two exposure groups were not well balanced. The largest imbalance by a wide margin was body mass index (SMD −1.29 in younger and −1.11 in older patients). In younger patients, creatinine, systolic blood pressure, hypertension, hemodialysis, Glasgow Coma Scale, hemoglobin and APACHE II score were also imbalanced; in older patients, cerebrovascular disease or dementia was the most notable categorical imbalance. These imbalances are the reason the primary analysis is adjusted for body mass index, sex and comorbidity, and the reason the residual-confounding limitation is stated explicitly in the Discussion.

Supplementary information S9. ICU all cause mortality

Group	Sample		Survival Probability at Specified Time Points (%)				Log-rank p (Non-low vs Low within stratum)
	n	Events n (%)	7-day (95% CI)	14-day (95% CI)	28-day (95% CI)	60-day (95% CI)	
**Younger Patients**							
Non-low thoracic muscle area	195	75 (38.5)	88.0 (82.2–92.1)	77.8 (69.6–84.0)	48.6 (37.4–58.9)	23.1 (12.7–35.3)	0.790
Low thoracic muscle area	68	29 (42.6)	75.9 (62.5–85.1)	64.6 (49.8–76.1)	49.0 (33.6–62.7)	40.2 (23.9–56.0)	
**Older Patients**							
Non-low thoracic muscle area	264	113 (42.8)	85.9 (80.5–89.9)	68.9 (61.4–75.2)	43.7 (34.7–52.3)	9.2 (3.7–17.7)	0.874
Low thoracic muscle area	80	40 (50.0)	84.6 (73.8–91.2)	69.3 (55.6–79.4)	39.2 (24.7–53.4)	13.5 (3.4–30.4)	

Survival probabilities are presented as percentages with 95% confidence intervals computed by Greenwood's formula on the log–log scale. Median survival is the smallest time at which the Kaplan–Meier estimate drops to ≤ 0.50. Within each age stratum, the low thoracic muscle area group was compared with the non-low thoracic muscle area group using the log-rank (Mantel–Cox) test.

Abbreviations: ICU, intensive care unit; CI, confidence interval.

Supplementary information S10. Landmark Analysis: ICU Mortality by Early (≤7 days) vs Late (>7 days) Phase

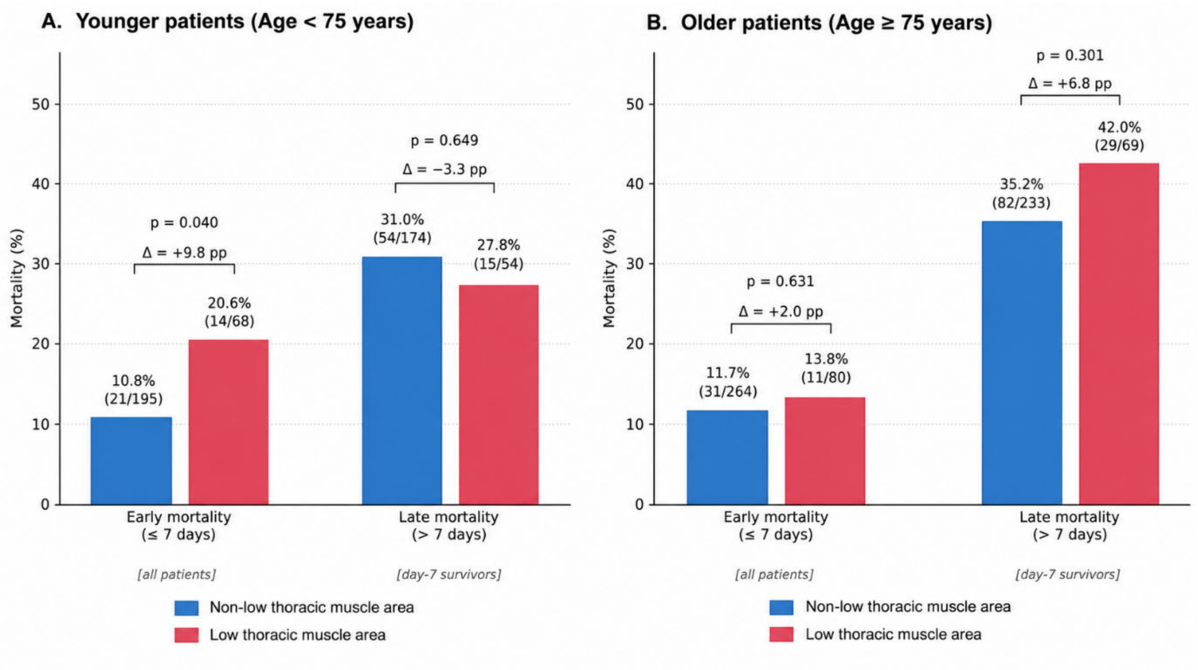

The left pair of bars represents early mortality (death within 7 days of ICU admission), calculated over all patients in each subgroup, while the right pair represents late mortality (death after day 7), calculated only among patients surviving to day 7 (a true landmark restriction). Blue bars denote non-low thoracic muscle area patients and red bars denote low thoracic muscle area patients. The numbers above each bar indicate the mortality percentage with the number of events over the at-risk denominator in parentheses. The bracketed annotation between paired bars displays the absolute between-group difference in percentage points (Δ pp) and the p-value from a Pearson chi-square test (Fisher's exact test substituted when any expected cell count was <5). A statistically significant difference ($p < 0.05$) is highlighted in red and marked with an asterisk.

Abbreviations: pp, percentage points; Δ, between-group difference; ICU, intensive care unit.

Supplementary information S11. Univariable and multivariable logistic regression analysis

Variable	Younger Patients (Age < 75 years)				Older Patients (Age ≥ 75 years)			
	Univariable		Multivariable		Univariable		Multivariable	
	OR (95% CI)	p-value	aOR (95% CI)	p-value	OR (95% CI)	p-value	aOR (95% CI)	p-value
**Composite Weaning Failure**								
Low thoracic muscle area	3.30 (1.76-6.21)	<0.001	3.63 (1.67-7.87)	0.001	1.70 (0.99-2.94)	0.055	1.68 (0.91-3.13)	0.100
Age (per year)	1.00 (0.97-1.02)	0.883	0.97 (0.95-1.01)	0.104	1.00 (0.96-1.05)	0.898	1.00 (0.96-1.05)	0.981
APACHE II score	1.04 (1.00-1.09)	0.062	1.05 (0.98-1.13)	0.147	1.01 (0.97-1.05)	0.732	1.03 (0.98-1.09)	0.194
PaO_2/FiO_2 ratio	1.00 (0.99-1.00)	0.051	1.00 (0.99-1.00)	0.028	1.00 (0.99-1.00)	0.005	0.99 (0.99-1.00)	0.004
GCS	1.01 (0.94-1.08)	0.849	1.08 (0.97-1.20)	0.163	1.03 (0.96-1.09)	0.448	1.07 (0.98-1.16)	0.146
Vasopressor use	2.33 (1.37-3.96)	0.002	3.05 (1.63-5.70)	<0.001	1.40 (0.89-2.21)	0.148	1.21 (0.74-1.99)	0.450
Body mass index	0.93 (0.88-0.98)	0.010	0.93 (0.87-1.00)	0.054	0.96 (0.91-1.02)	0.162	0.98 (0.92-1.05)	0.633
Male sex	0.91 (0.49-1.68)	0.752	0.67 (0.33-1.36)	0.263	0.86 (0.54-1.39)	0.540	0.91 (0.54-1.54)	0.727
Diabetes mellitus	0.93 (0.55-1.60)	0.806	1.44 (0.74-2.80)	0.280	0.78 (0.50-1.24)	0.302	0.70 (0.43-1.14)	0.151
COPD	5.20 (1.10-24.61)	0.038	2.53 (0.46-13.83)	0.284	0.66 (0.30-1.45)	0.303	0.56 (0.24-1.30)	0.176
Chronic heart failure	2.18 (0.20-24.40)	0.526	3.96 (0.27-58.78)	0.317	2.09 (0.69-6.40)	0.195	1.72 (0.53-5.53)	0.365
Chronic kidney disease	0.61 (0.23-1.60)	0.311	0.42 (0.13-1.38)	0.153	0.78 (0.42-1.44)	0.434	0.62 (0.32-1.23)	0.170
**Extubation Failure**								
Low thoracic muscle area	1.88 (1.02-3.43)	0.042	2.61 (1.21-5.63)	0.015	1.75 (1.00-3.05)	0.048	1.82 (0.96-3.44)	0.066
Age (per year)	0.98 (0.96-1.01)	0.158	0.96 (0.93-0.99)	0.015	1.01 (0.96-1.05)	0.759	1.00 (0.96-1.05)	0.973
APACHE II score	1.03 (0.99-1.08)	0.169	1.03 (0.96-1.10)	0.432	1.01 (0.97-1.05)	0.651	1.02 (0.96-1.07)	0.513
PaO_2/FiO_2 ratio	0.99 (0.99-1.00)	0.007	0.99 (0.99-1.00)	0.005	0.99 (0.99-1.00)	0.005	1.00 (0.99-1.00)	0.012
GCS	0.98 (0.91-1.05)	0.574	1.01 (0.90-1.13)	0.885	1.01 (0.95-1.09)	0.692	1.04 (0.95-1.14)	0.388
Vasopressor use	3.10 (1.77-5.43)	<0.001	3.85 (2.01-7.36)	<0.001	1.78 (1.10-2.89)	0.019	1.56 (0.93-2.63)	0.093
Body mass index	0.98 (0.93-1.03)	0.443	0.97 (0.91-1.04)	0.449	0.98 (0.92-1.04)	0.483	1.01 (0.94-1.08)	0.848
Male sex	0.81 (0.43-1.54)	0.526	0.67 (0.32-1.38)	0.274	0.75 (0.46-1.24)	0.264	0.72 (0.41-1.25)	0.243
Diabetes mellitus	0.72 (0.41-1.28)	0.269	1.10 (0.55-2.20)	0.787	1.14 (0.70-1.85)	0.596	0.98 (0.58-1.65)	0.937
COPD	3.50 (0.99-12.34)	0.051	1.60 (0.38-6.68)	0.521	0.77 (0.33-1.80)	0.540	0.78 (0.31-1.94)	0.596
Chronic heart failure	3.82 (0.34-42.82)	0.277	6.07 (0.36-103.49)	0.212	1.58 (0.53-4.68)	0.409	1.16 (0.37-3.68)	0.796
Chronic kidney disease	0.65 (0.22-1.87)	0.423	0.52 (0.15-1.84)	0.309	1.19 (0.63-2.25)	0.584	1.03 (0.52-2.07)	0.923
**Tracheostomy**								
Low thoracic muscle area	2.06 (1.09-3.89)	0.026	1.14 (0.50-2.58)	0.757	2.23 (1.22-4.10)	0.010	2.34 (1.15-4.75)	0.019
Age (per year)	1.01 (0.98-1.04)	0.458	1.01 (0.98-1.05)	0.502	0.99 (0.94-1.04)	0.682	0.99 (0.94-1.05)	0.761
APACHE II score	1.00 (0.96-1.05)	0.861	0.96 (0.89-1.03)	0.235	0.98 (0.94-1.03)	0.467	1.01 (0.95-1.08)	0.754
PaO_2/FiO_2 ratio	1.00 (1.00-1.01)	0.042	1.00 (1.00-1.01)	0.060	1.00 (1.00-1.01)	0.494	1.00 (1.00-1.00)	0.763
GCS	0.99 (0.91-1.07)	0.749	0.96 (0.85-1.08)	0.501	1.03 (0.95-1.11)	0.539	1.05 (0.94-1.17)	0.384
Vasopressor use	1.76 (0.98-3.17)	0.060	2.12 (1.07-4.20)	0.031	0.88 (0.50-1.55)	0.656	0.99 (0.53-1.83)	0.967
Body mass index	0.89 (0.83-0.96)	0.001	0.88 (0.80-0.97)	0.008	0.97 (0.90-1.04)	0.363	1.01 (0.93-1.10)	0.783
Male sex	1.93 (0.88-4.23)	0.103	1.68 (0.71-3.96)	0.235	0.88 (0.49-1.57)	0.665	1.07 (0.56-2.04)	0.828
Diabetes mellitus	0.93 (0.50-1.72)	0.821	1.52 (0.72-3.17)	0.269	0.81 (0.46-1.43)	0.471	0.81 (0.44-1.49)	0.500
COPD	5.44 (1.53-19.31)	0.009	7.26 (1.68-31.49)	0.008	0.99 (0.38-2.53)	0.975	0.82 (0.29-2.32)	0.706
Chronic heart failure	NE	—	NE	—	1.55 (0.47-5.10)	0.474	1.80 (0.48-6.75)	0.384
Chronic kidney disease	0.50 (0.14-1.79)	0.291	0.67 (0.16-2.74)	0.574	0.13 (0.03-0.55)	0.006	0.13 (0.03-0.56)	0.006
**Prolonged MV (>21 days)**								
Low thoracic muscle area	3.35 (1.32-8.51)	0.011	3.79 (1.14-12.59)	0.030	1.26 (0.48-3.33)	0.643	0.96 (0.32-2.89)	0.949

Variable	Younger Patients (Age < 75 years)				Older Patients (Age ≥ 75 years)			
	Univariable		Multivariable		Univariable		Multivariable	
	OR (95% CI)	p-value	aOR (95% CI)	p-value	OR (95% CI)	p-value	aOR (95% CI)	p-value
Age (per year)	1.00 (0.96-1.04)	0.935	0.98 (0.92-1.03)	0.424	1.01 (0.93-1.09)	0.801	1.01 (0.94-1.09)	0.773
APACHE II score	1.07 (0.99-1.15)	0.094	1.08 (0.96-1.21)	0.200	1.02 (0.95-1.09)	0.679	1.05 (0.96-1.16)	0.278
PaO_2/FiO_2 ratio	0.99 (0.99-1.00)	0.126	0.99 (0.99-1.00)	0.167	1.00 (0.99-1.00)	0.177	0.99 (0.99-1.00)	0.138
GCS	1.01 (0.89-1.14)	0.906	1.10 (0.91-1.33)	0.322	1.06 (0.93-1.20)	0.392	1.13 (0.96-1.33)	0.154
Vasopressor use	2.05 (0.80-5.22)	0.132	1.71 (0.57-5.15)	0.337	1.32 (0.56-3.09)	0.523	1.08 (0.43-2.71)	0.868
Body mass index	0.91 (0.81-1.01)	0.082	0.95 (0.84-1.09)	0.475	0.93 (0.83-1.04)	0.220	0.93 (0.82-1.06)	0.263
Male sex	0.85 (0.29-2.46)	0.763	0.66 (0.19-2.31)	0.517	0.77 (0.32-1.85)	0.565	0.67 (0.26-1.75)	0.410
Diabetes mellitus	0.58 (0.20-1.65)	0.305	0.91 (0.26-3.15)	0.881	0.79 (0.32-1.93)	0.605	0.86 (0.33-2.20)	0.748
COPD	11.50 (3.14-42.09)	<0.001	6.32 (1.26-31.67)	0.025	1.48 (0.41-5.33)	0.546	1.26 (0.32-5.04)	0.742
Chronic heart failure	NE	—	NE	—	0.94 (0.12-7.56)	0.957	0.64 (0.07-5.60)	0.689
Chronic kidney disease	0.57 (0.07-4.51)	0.594	0.30 (0.02-3.83)	0.356	0.75 (0.22-2.64)	0.658	0.59 (0.15-2.25)	0.438
**ICU Mortality**								
Low thoracic muscle area	1.10 (0.60-2.02)	0.762	1.35 (0.60-3.04)	0.463	1.14 (0.66-1.95)	0.644	1.13 (0.57-2.22)	0.727
Age (per year)	1.02 (0.99-1.04)	0.206	0.99 (0.96-1.03)	0.572	1.01 (0.96-1.05)	0.768	1.01 (0.96-1.06)	0.732
APACHE II score	1.06 (1.01-1.11)	0.010	1.11 (1.03-1.20)	0.004	1.04 (1.01-1.09)	0.026	1.06 (1.00-1.13)	0.038
PaO_2/FiO_2 ratio	0.99 (0.99-1.00)	<0.001	0.99 (0.99-1.00)	<0.001	0.99 (0.99-1.00)	<0.001	0.99 (0.99-1.00)	<0.001
GCS	1.03 (0.96-1.11)	0.427	1.17 (1.04-1.32)	0.009	0.99 (0.93-1.05)	0.706	1.05 (0.95-1.16)	0.322
Vasopressor use	4.64 (2.62-8.19)	<0.001	6.08 (3.06-12.09)	<0.001	5.68 (3.46-9.35)	<0.001	4.97 (2.90-8.50)	<0.001
Body mass index	0.99 (0.94-1.05)	0.756	0.95 (0.88-1.02)	0.170	0.95 (0.90-1.01)	0.102	0.99 (0.91-1.06)	0.716
Male sex	0.62 (0.33-1.16)	0.138	0.51 (0.24-1.10)	0.087	1.16 (0.72-1.87)	0.553	1.24 (0.69-2.23)	0.476
Diabetes mellitus	1.00 (0.57-1.74)	0.998	1.08 (0.53-2.22)	0.832	0.82 (0.51-1.30)	0.393	0.58 (0.34-1.02)	0.057
COPD	2.09 (0.62-7.06)	0.236	0.42 (0.10-1.82)	0.244	0.67 (0.30-1.49)	0.325	0.51 (0.20-1.28)	0.151
Chronic heart failure	NE	—	NE	—	2.47 (0.81-7.56)	0.112	1.98 (0.56-7.04)	0.291
Chronic kidney disease	1.24 (0.48-3.22)	0.655	0.61 (0.17-2.23)	0.453	1.27 (0.69-2.32)	0.448	0.73 (0.35-1.52)	0.398

Interaction p (Low thoracic muscle mass × Age group): Composite Weaning Failure, 0.061; Extubation Failure, 0.436; Tracheostomy, 0.805; Prolonged MV (>21 days), 0.089; ICU Mortality, 0.685.

Multivariable models were adjusted for all covariates listed in the table. Each interaction p value is from a single pooled model containing low thoracic muscle area, age group, their product term and the same covariates.Analyses were restricted to complete cases (young n = 233; elderly n = 306; total n = 539) because body mass index was missing in 68 of 607 patients. Univariable estimates are reported on the same complete-case sample as the multivariable models so that crude and adjusted estimates are directly comparable. Events per stratum: composite weaning failure 112/144; extubation failure 81/100; tracheostomy 61/64; prolonged MV 20/23; ICU mortality 87/132 (young/elderly).

NE, not estimable. Chronic heart failure was present in only 3 younger patients, none of whom underwent tracheostomy, required prolonged mechanical ventilation, or survived the ICU, producing complete separation; these cells are therefore not estimable. Excluding chronic heart failure from the young-stratum models left the sarcopenia estimates essentially unchanged (composite 3.63, extubation failure 2.59, tracheostomy 1.16, prolonged MV 3.81, ICU mortality 1.33).

Abbreviations: OR, odds ratio; aOR, adjusted odds ratio; CI, confidence interval; APACHE II, Acute Physiology and Chronic Health Evaluation II; PaO2/FiO2, ratio of partial pressure of arterial oxygen to fraction of inspired oxygen; GCS, Glasgow Coma Scale; COPD, chronic obstructive pulmonary disease; MV, mechanical ventilation; ICU, intensive care unit.

Supplementary information S12. Dose–response of composite weaning failure under alternative scalings of thoracic muscle mass

Panel A. Absolute thoracic muscle area, fully adjusted

Metric	Younger (<75 y)	Older (≥75 y)
Failure rate, Q1 (lowest)	69.5%	57.4%
Failure rate, Q2	46.8%	50.6%
Failure rate, Q3	46.9%	44.9%
Failure rate, Q4 (highest)	33.3%	34.4%
Q1 vs Q4, aOR (95% CI)	4.79 (1.73–13.30)	2.53 (1.08–5.95)
Q1 vs Q4, P	0.003	0.033
Per-quartile increase, OR (P for trend)	0.618 (0.004)	0.743 (0.033)

Panel B. Muscle area indexed to height squared, fully adjusted

Metric	Younger (<75 y)	Older (≥75 y)
Failure rate, Q1 (lowest)	64.7%	59.7%
Failure rate, Q2	48.8%	52.3%
Failure rate, Q3	38.0%	42.7%
Failure rate, Q4 (highest)	38.9%	35.1%
Q1 vs Q4, aOR (95% CI)	2.48 (0.90–6.83)	3.12 (1.28–7.63)
Q1 vs Q4, P	0.080	0.012
Per-quartile increase, OR (P for trend)	0.710 (0.042)	0.695 (0.012)

The age contrast in the dose–response relationship is therefore not robust either to the covariate set or to the scaling of muscle mass; on a body-size-normalized scale with full adjustment the gradient is in fact clearer in older than in younger patients. These are exploratory analyses: the quartile models contain 14 parameters against 112 and 144 outcome events in the two strata, so individual quartile estimates are imprecise.
